# A new method for augmenting short time series, with application to pain events in sickle cell disease

**DOI:** 10.1371/journal.pcbi.1014389

**Published:** 2026-06-12

**Authors:** Kumar Utkarsh, Nirmish R. Shah, Tanvi Banerjee, Daniel M. Abrams

**Affiliations:** 1 Department of Engineering Sciences and Applied Mathematics, Northwestern University, Evanston, Illinois, United States of America; 2 Department of Medicine, Duke University, Durham, North Carolina, United States of America; 3 Department of Computer Science and Engineering, Wright State University, Dayton, Ohio, United States of America; 4 Northwestern Institute for Complex Systems, Northwestern University, Evanston, Illinois, United States of America; 5 Department of Physics and Astronomy, Northwestern University, Evanston, Illinois, United States of America; Xinjiang Technical Institute of Physics and Chemistry, CHINA

## Abstract

Researchers across different fields, including but not limited to ecology, biology, and healthcare, often face the challenge of sparse data. Such sparsity can lead to uncertainties, estimation difficulties, and potential biases in modeling. Here we introduce a novel data augmentation method that combines multiple sparse time series datasets when they share similar statistical properties, thereby improving parameter estimation and model selection reliability. We demonstrate the effectiveness of this approach through validation studies comparing Hawkes and Poisson processes, followed by application to subjective pain dynamics in patients with sickle cell disease (SCD), a condition affecting millions worldwide, particularly those of African, Mediterranean, Middle Eastern, and Indian descent.

## Author summary

When studying health conditions like sickle cell disease, we often face a frustrating challenge: individual patient datasets are too small or sparse to draw reliable conclusions. We developed a method to overcome this by combining data from multiple patients who show similar patterns, effectively treating them as different snapshots of the same underlying process. We tested this approach on pain event data from sickle cell disease patients, where understanding pain patterns is crucial for improving care but individual records are often incomplete. Our method revealed that pain events in most patients follow a “self-exciting” pattern, something that couldn’t be confidently determined from individual patient data alone. This technique could help researchers in many fields where data is scarce but understanding temporal patterns is essential, from ecology to healthcare, enabling more reliable insights from limited observations.

## Introduction

Patient-reported data (including data from wearable devices) has gained increasing prominence in healthcare settings recently [1–9]. This data, however, is often characterized by inherent variability, including fluctuations in reporting frequency and content, which complicates analysis. Moreover, the temporal dynamics associated with health events, including symptom fluctuations or treatment responses, pose a multitude of analytical challenges. Stochastic models provide tools for analyzing such data, when sufficiently abundant, allowing researchers and clinicians to learn useful information from observed patterns over time [10–12]. However, attempting to draw conclusions from too narrow a set of observations can lead to overfitting, unreliable estimates, and difficulties in generalizing [13–15].

To address these issues, researchers have developed methodologies such as Bayesian approaches, which incorporate prior information and uncertainty [16], and hierarchical models that pool data across related groups to enhance estimates for conditions with sparse data [17]. Additionally, augmentation techniques like bootstrapping and space-filling algorithms allow for the generation of synthetic data points, improving the robustness of statistical analyses [18–21]. However, little has been done on sparse sampling in the context of systems with inherent temporal correlations and dependence structure. In this paper, we develop a new method that has broad applicability to sparsely sampled data from dynamical processes, and we focus in particular on testing the method in the real-world context of data capturing pain events in patients with sickle cell disease. Unlike synthetic augmentation approaches, which generate artificial data points, our method pools real observations across statistically similar datasets. In particular, given a collection of sparse time series suspected to share underlying dynamics, our augmentation strategy improves both model discrimination and parameter recovery compared to single-series analysis, with performance gains that vanish when series are drawn from unrelated processes.

## Materials and methods

### Model and assumptions

Our approach is motivated by collections of datasets that have irregular sampling and limited length, but where at least a subset of the collection may be well explained by a single model. This is true for our example collection of pain events in patients with SCD.

In particular, the frequency of early hospital readmission in SCD patients (almost 90% readmitted within 30 days [22,23]) suggests that pain events may naturally cluster temporally. Therefore, we treat the occurrence of significant pain events as a self-exciting process, meaning that patients are at elevated risk for a subsequent event immediately after one occurs. This type of process was first described mathematically by Alan G. Hawkes in the context of modeling seismic activity [24–26], where earthquakes trigger aftershocks. The analogy extends naturally to SCD, where initial obstructions in blood flow result in inflammatory responses that may precipitate subsequent pain episodes. Similar dynamics have been reported in criminal activities [27] and financial markets [28]. We thus treat the occurrence of pain events as a *Hawkes process*.

A Hawkes process is a counting process {N(t)|t≥0} with an associated history $ℋ(t):=\{t_{i}|t_{i}<t\}$, where $t_{i}$ is the time of $i^{th}$ event, and a conditional intensity function λ(t|ℋ(t)) of the form


$$\begin{array}{c} \lambda (t|ℋ(t))=\lambda_{0}(t)+\sum_{i:t>t_{i}}\Phi (t-t_{i}), \end{array}$$ (1)

where $\lambda_{0}>0$ is the *baseline intensity* and Φ≥0 is a monotonically decreasing function referred as the *memory kernel*.

In such a process, the occurrence of an event increases the likelihood of a subsequent event for some time after the initial arrival (with this “memory timescale” set by the decay rate of Φ). Henceforth, the terms “conditional intensity function” and “intensity function” will be used interchangeably, and λ(t)≡λ(t|ℋ(t)).

We further simplify the Hawkes process model by assuming a constant baseline intensity $\lambda_{0}(t)=\lambda_{0}$ and an exponential memory kernel $\Phi (t-t_{i})=\exp[-\delta (t-t_{i})]$ with δ>0. We choose the exponential kernel for three reasons: (a) it provides analytical tractability, allowing closed-form likelihood computation; (b) it gives clear parameter interpretability with $\delta^{-1}$ representing a characteristic memory timescale; and (c) it is physiologically plausible, as inflammatory cascades likely exhibit exponential decay kinetics.

**Fig 1:**
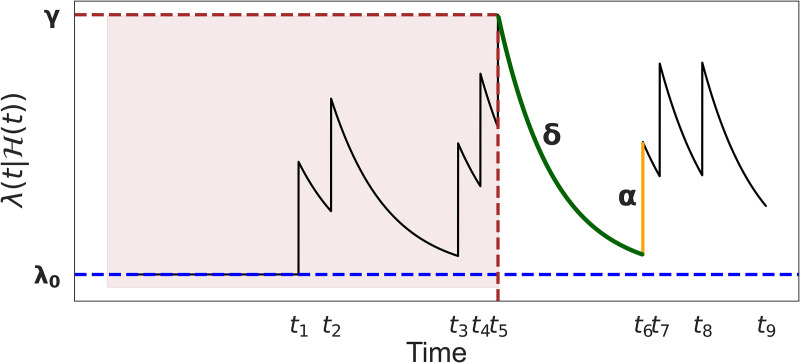
Visual guide to shifted Hawkes process parameters and intensity dynamics. Characterization of the parameters introduced in Eq. (2) (see also Table 1). The peaks represent event arrivals in real-time. The shaded area represents the history not captured in the observed data. In this example, the observable *t* = 0 is *t*_5_. The blue horizontal dashed line shows the value of the baseline intensity $\lambda_{0}$ and the red horizontal dashed line shows the initial intensity value γ. The amplitude of impact α is depicted by the length of the vertical yellow line segment, whereas the decay rate δ characterizes the exponential decay curves, like the one shown in green.

One common assumption in the use of Hawkes models is that the process is observed from its onset. However, in practice, data may be collected during an arbitrary interval in the middle a longer process—this is the case for our SCD datasets, which begin months to years after the onset of SCD symptoms. Keeping that in mind, we introduce an additional compensatory term to capture past events not recorded in the dataset, $(\gamma -\lambda_{0})e^{-\delta t}$. This yields our proposed model intensity function


$$\begin{array}{c} \lambda (t)=\lambda_{0}+\alpha \sum_{i:t>t_{i}}e^{-\delta (t-t_{i})}+(\gamma -\lambda_{0})e^{-\delta t}, \end{array}$$ (2)

where $\lambda_{0}>0,\alpha \geq 0,\delta >0,$ and γ≥0 are constants and $t_{i}$ is the time of $i^{th}$ event. A description of each parameter can be found in Table 1 and parameters are visualized in Fig 1.

**Table 1:** Description of model parameters introduced in Eq. (2) (see also Fig 1).

Parameter	Description	Units
$\lambda_{0}$	Baseline intensity value for underlying homogeneous Poisson process.	$\left[T^{-1}\right]$
α	Amplitude of impact of an individual event arrival on intensity.	$\left[T^{-1}\right]$
δ	Rate of decay to baseline intensity ($\delta^{-1}$ sets memory length).	$\left[T^{-1}\right]$
γ	The intensity measurement recorded at the initiation of the data collection period.	$\left[T^{-1}\right]$

We note that this model is designed for time series datasets that capture an event at *t* = 0, which serves as the reference point for the initial condition γ. The key parameters dictating model behavior are $\lambda_{0}$, α and δ, whereas γ should be seen as a correction factor to compensate for the effects of past history not captured in the dataset.

### Model fitting and selection

Before fitting to data, we wish to establish what would characterize a “successful” model. In particular, we would like to choose a reasonable null model against which to test, and we hope to deduce exactly how much data is necessary to distinguish among models.

Substituting α=0 and $\gamma =\lambda_{0}$ into Eq. (2) yields a homogeneous Poisson process (henceforth referred to simply as “a Poisson process”), but this is just a special case of the Hawkes model where memory effects are absent. The same limiting behavior arises when δ→∞, meaning memories disappear instantaneously after each event. Conversely, the Hawkes model can be seen as an extension over a baseline Poisson model with a self-exciting memory kernel. For this reason, the Poisson process is a natural candidate for a null model. In addition to the fact that it represents a simple and widely used framework for modeling point processes, we can assess whether the added complexity of the Hawkes process results in a statistically significant improvement.

We fit model parameters using maximum likelihood estimates, where likelihood for a dataset $\{t_{i}{\}}_{i=1}^{N}$ is given by


$$\begin{array}{c} L(\theta |t_{1},t_{2},...,t_{N})=\left(\prod_{i=1}^{N}\lambda (t_{i})\right)e^{-\int_{0}^{t_{N}}\lambda (s)ds}. \end{array}$$ (3)

For the Hawkes model, if we know the complete history (as in simulations), the process starts at baseline intensity $\gamma =\lambda_{0}$, and is defined by


$$\begin{array}{c} \lambda (t)=\lambda_{0}+\alpha \sum_{i:t>t_{i}}e^{-\delta (t-t_{i})}. \end{array}$$ (4)

We use the Akaike Information Criterion (AIC) for the comparison, as it provides a theoretically grounded balance between a model’s goodness of fit and complexity [29] (though we note that there are potential problems with the use of information criteria in dynamical systems [30]). Our candidate model has four degrees of freedom ($\lambda_{0},\alpha ,\delta ,\gamma$), while the null model has just one ($\lambda_{\text{P}}$). Since these models are nested, with the Poisson model being a simpler version of the Hawkes model, differences in complexity can make it challenging to recover the true model, especially with limited data.

**Fig 2:**
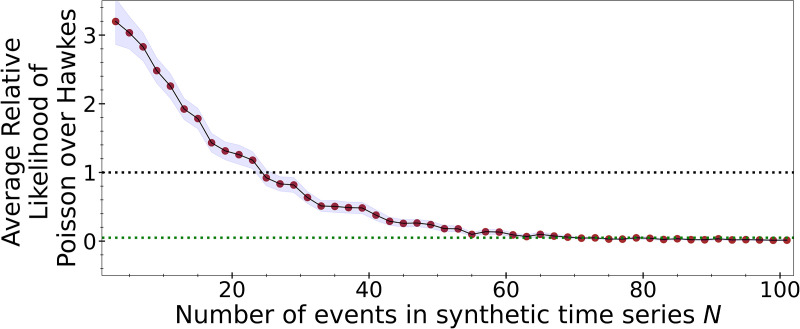
Minimum dataset size required for reliable Hawkes vs. Poisson model discrimination. Number of data points needed to distinguish Hawkes model from Poisson. The black dashed line is for basic preference (ℒ=1), whereas the green dashed line is for 95% confidence (ℒ=0.05). For each *N*, we calculate the relative likelihood 50 times. The averages are calculated, and denoted by the red markers. The purple-shaded region denotes the 95% confidence interval. The likelihood values and critical *N* depend on the parametric choices. We use ($\lambda_{0}$, α, δ)=(1, 3, 6).

To illustrate, we simulate multiple time series of varying lengths using Eq. (4). Fig 2 shows that AIC often reflects greater evidence for the Poisson model for shorter time series, indicating a threshold length (dependent on parameter choices) below which this approach fails to detect the true dependency structure.

This aligns with the principle that AIC generally performs better with larger sample sizes, where the trade-off between fit and complexity becomes clearer [31]. A version of Fig 2 using the corrected AIC (AICc) [32] is provided in S3 Appendix. Corrections for finite data do not resolve the sparse data problem, they have the opposite impact of making it a greater challenge.

### Data augmentation

Our idea for augmenting sparse datasets is as follows: (1) we test datasets, pairwise, for statistical similarity; (2) we replace each individual dataset with an ensemble of those shown to be similar; and (3) we fit model parameters to each full ensemble, treating it as composed of disconnected excerpts drawn from a single process.

Concretely, we use the two-sample Kolmogorov-Smirnov (KS) test to assess pairwise similarity between datasets. The KS test is a nonparametric method that compares the empirical cumulative distribution functions of two independent samples, making no assumption about the functional form of the underlying distribution. It is sensitive to differences in both the location and shape of the distribution, and is computationally trivial to apply pairwise across a collection of many series.

**Fig 3:**
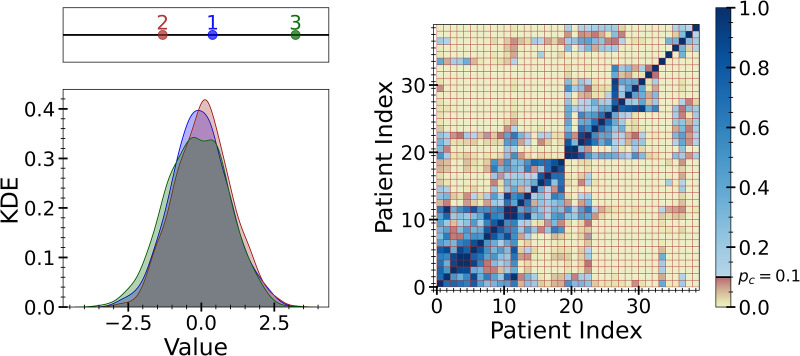
KS test demonstrates non-transitive similarity and identifies matchable patient pairs. (*Left*) Two-sample KS test for three normal distributions (1, 2, and 3) with slightly different means and variances (colored blue, red, and green, respectively). The three *p*-values for (1 vs. 2), (1 vs. 3), and (2 vs. 3) are 0.03, 0.02, and 10^−5^, respectively. This example demonstrates the non-transitive nature of the test: the mutual distances between the dots in the upper panel are in proportion to the mutual KS statistics; 1 is similar to both 2 and 3, whereas 2 and 3 are far apart and are thus dissimilar. (*Right*) Similar patients in our collection of datasets using the two-sample KS test (0.1 significance level). Blue shades for matched pairs of patients and yellow-pink shades for unmatched.

By applying the KS test, we can systematically group datasets with comparable characteristics, enabling a collective analysis. The null hypothesis for this test is that two samples come from the same distribution. The test statistics and sample sizes are used to calculate the *p*-values for the test, and the hypothesis is accepted if this *p*-value exceeds our set threshold $p_{c}$. An example of its application is shown in Fig 3.

We note that, since the KS test compares samples drawn from distributions, it cannot be applied directly to entire time series. To address this in the context of our patient data, we instead compare the distributions of interarrival times between events, which capture the underlying temporal structure. We characterize the sensitivity and limitations of this criterion systematically in S4 Appendix.

Once similar datasets are identified, we define a “collective likelihood” that integrates information across these matched groups, enhancing the reliability of model selection in contexts where individual datasets are too sparse for robust analysis. The following provides a step-by-step description of this strategy, applied to a collection of small or sparse time series where model selection might otherwise be unreliable:

*Step 1:* Consider a collection of *m* time series datasets $𝒞=\{t_{i}^{j}:i=1\ldots n_{j},j=1\ldots m\}$. Compute the set of interarrival times $\Delta 𝒞=\{\Delta t_{i}^{j}:i=1\ldots n_{j}-1,j=1\ldots m\}$.
*Step 2:* Calculate the *p*-values using a two-sample KS test for each pair of datasets of interarrival times and define the matrix 𝐏={p(i,j):i,j=1…m}. Those with *p*-values below a given threshold $p_{c}$ are taken to be similar.
*Step 3:* We define the collective likelihood ($\tilde{L}$) of a model for dataset *j* as
  $$\begin{array}{c} {\tilde{L}}_{j}=L_{j}\prod_{i:{𝐏}_{ij}\geq p_{c},i\neq j}L_{i}, \end{array}$$ (5)
  where $L_{i}$ is the individual likelihood of a given model for dataset *i* and $p_{c}$ is a similarity threshold. This way, we consider the similar datasets to be different realizations of the same process. The neighbourhood 𝒩(j) for dataset *j* is defined as
  $$\begin{array}{c} 𝒩(j)=\{\;i\neq j|{𝐏}_{ij}\geq p_{c}\;\}. \end{array}$$
  No transitive closure is applied, so each dataset receives an independent augmentation group reflecting only its direct pairwise similarities.
*Step 4:* We calculate the best-fit parameters and AIC values for each model using the collective likelihood. We use these for model selection.

Note that this aggregation approach is non-transitive due to the non-transitivity of the KS test. If datasets A and B are statistically similar, and B and C are as well, it need not be true that A and C are. This point is illustrated through Fig 3 (*left*) and Step 3. Thus, the model parameters ultimately fitted to A, B, and C may all end up different. The dependence of neighbourhood size on threshold choice is characterized in S4 Appendix.

To understand the statistical sense behind the construction of the collective likelihood in Eq. (5), note that for a single series the likelihood is simply a product of probability densities over observed interarrival times under a given parameter vector θ. The collective likelihood extends this product directly to *k* matched series, with the same θ appearing in every factor—equivalent to treating the matched series as disjoint entities of a single longer realization of the same process. This is strictly stronger than hierarchical or partial-pooling formulations [17], which allow parameters to vary across group members under a shared hyperprior. Here, the implicit assumption is exact exchangeability: any series in the augmentation group could have been observed in place of any other, because all are governed by the same generative mechanism. This assumption is what makes the KS-based grouping step essential: it is the mechanism by which we verify, before computing the collective likelihood, that the exchangeability condition is approximately satisfied.

## Results

### Model analysis and method verification

#### Differentiating Hawkes from Poisson.

We consider two processes, a Poisson process with $\lambda_{\text{P}}=2$ and a Hawkes process (Eq. (4)) with comparable stationary behavior. Specifically we choose parameters such that the expected value of its equilibrium intensity $𝔼[\lambda (t)]=\lambda_{0}(1-\alpha /\delta {)}^{-1}$ is 2 as well (see S1 Appendix for a brief derivation of this formula and S2 Appendix for a brief discussion of parameter identifiability issues).

The goal of this numerical experiment is to test whether the proposed data augmentation strategy improves model selection by enhancing the ability to distinguish between the two processes. Specifically, we evaluate the AIC differences between the Poisson and Hawkes models fitted to $N_{aug}=10$ original single-series datasets with $N_{e}=30$ events each and a single augmented datasets for each model class.

**Fig 4:**
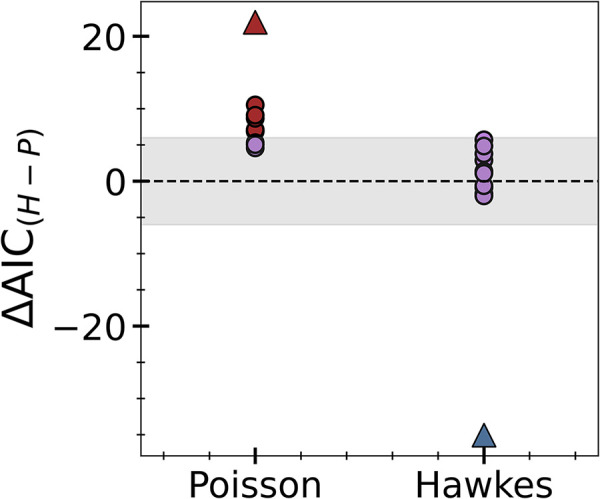
Data augmentation shifts preference from inconclusive to confident model selection. We generate 20 synthetic times series, 10 from a Poisson process and 10 from a Hawkes process, then examine the relative statistical support $\Delta {AIC}_{\left(H-P\right)}=AIC(H)-AIC(P)$ for each model before and after augmentation. Each single-series realization is shown as a colored circle, and augmented datasets are shown as larger triangles with the same color scheme. Blue markers indicate a preference for the Hawkes model, red markers indicate a preference for the Poisson model, and purple markers correspond to inconclusive cases. Points above the dashed line correspond to a preference for the Poisson model, while points below correspond to a preference for the Hawkes model. Here $N_{aug}=10$ and $N_{e}=30$ (events per time series). Regions outside the grey band correspond to model support with >95% confidence. Parameters: ($\lambda_{0}=1$, α=2, δ=3.5) and $\lambda_{P}=7/3$.

The results are summarized in Fig 4, where ΔAIC values are shown for single-series (colored circles) and augmented datasets (larger triangles). Blue markers indicate a preference for the Hawkes model, red markers indicate a preference for the Poisson model, and purple markers correspond to inconclusive cases. The gray shading in the figure represents the inconclusive region, corresponding to results within the 95% confidence interval where neither model is clearly preferred. The boundary for this inconclusive region can be derived from the relationship between ΔAIC and relative likelihoods ${ℒ}_{relative}=\exp\left(-\Delta \text{AIC}/2\right)$. At the 95% confidence level this yields $\Delta {\text{AIC}}^{\text{(crit)}}=-2\ln(0.05)\approx 6$ (so any |ΔAIC|>rsim6 lies outside the inconclusive region and indicates strong evidence favoring one model over the other).

Augmenting datasets consistently moves results outside the inconclusive region for both processes, demonstrating that the strategy does indeed enable more robust model selection.

#### Parameter estimation for augmented datasets.

**Fig 5:**
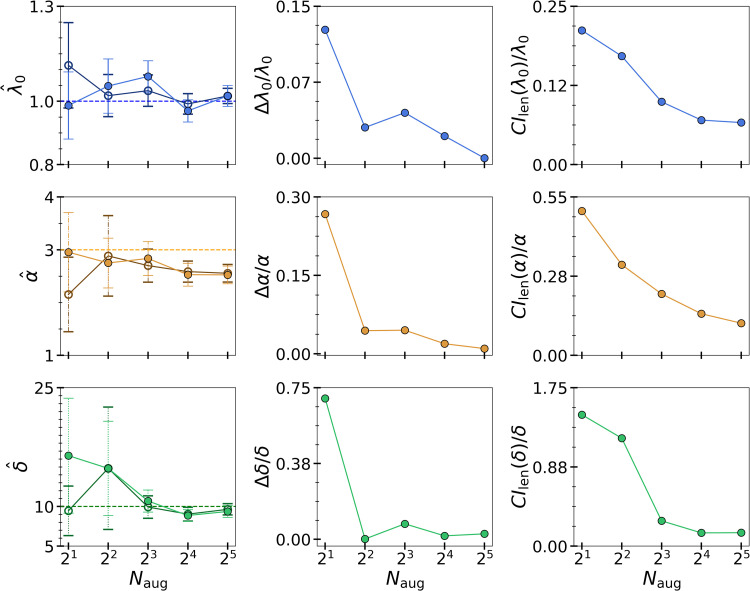
Augmented sparse series recover parameters comparably to equivalent-length continuous data. Comparison of parameter estimation performance between our data augmentation strategy (filled markers) and single continuous time-series (hollow markers) of equivalent total length $N_{e}N_{aug}$. All panels plot results versus $N_{aug}$ (number of augmented series), where each augmented series contains $N_{e}$ events. Top row: $\lambda_{0}$; middle row: α; bottom row: δ. Left column: estimated parameter values, where horizontal dashed lines indicate true values ($\lambda_{0}=1$, α=3, δ=10), markers represent means over 30 trials, and error bars show standard deviations. Center column: relative error between the two approaches, defined as $|{\hat{\theta }}_{full}-{\hat{\theta }}_{aug}|/\theta_{true}$, where ${\hat{\theta }}_{full}$ and ${\hat{\theta }}_{aug}$ are parameter estimates from the full series and augmented strategy, respectively. Right column: lengths of 95% confidence intervals, normalized by parameter values to facilitate comparison across different scales.

We wish to test the impact of our proposed data augmentation strategy on parameter estimation. To do so, we generate a collection of time series each consisting of $N_{e}$ events taken from an arbitary time interval in a Hawkes process. This mimics the situation often found in real-world datasets, where full histories are rarely available and only segments from a limited time interval are observed. We compare the best-fit estimators obtained from augmentation of $N_{aug}$ of these time series against the best-fit parameters derived from a single longer time series with a total of $N_{aug}N_{e}$ events. Fig 5 illustrates the effect of varying the number of augmentations while holding the number of events per series fixed at $N_{e}=16$.

As the figure shows, the data augmentation approach can recover parameters comparable to those obtained from an uninterrupted time series of equivalent total length. That is, our method can yield robust parameter estimates, effectively compensating for sparsity.

### Results for real-world data

#### Data.

Sickle cell disease (SCD) is a lifelong genetic disorder that affect hemoglobin, which is a carrier of oxygen in red blood cells (RBCs). In SCD, RBCs deform into “sickle” shapes, obstructing regular blood flow and causing potentially life-threatening problems. SCD affects more than 100,000 people in the US and 8 million people globally [33,34]. About 90% of acute care visits for SCD patients are associated with severe and frequent pain episodes. Understanding and modeling these pain episodes is crucial for improving patient care and treatment strategies, as they significantly impact both quality of life and healthcare costs [35].

**Fig 6:**
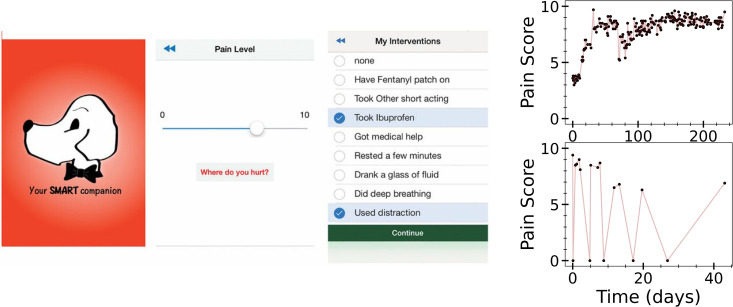
Real patient data. Sample screenshots from SMART app and typical patient time-series data collected using the app [36]. Note inter-patient variability, temporal irregularity, reporting fatigue, and other data quality challenges.

Our study employs data from our self-developed Sickle cell Mobile Application to Record symptoms via Technology, or SMART application [36–38]. Fig 6 shows two examples of patient-reported subjective pain data collected via this app. This data comes from a small cohort of 39 patients who were asked to report their pain levels every day. Although subjective pain reports may not fully correspond to physiological indicators, they remain central to SCD pain management because pain is inherently subjective and self-report is currently the only validated method for assessing pain severity (and has been shown to be a reliable indicators of clinical outcomes in SCD [39–42]). Among other things, the dataset includes pain levels and corresponding timestamps.

For our analysis, we treat the event times as the timestamps corresponding to only non-zero pain levels, where each reported pain level above zero constitutes an event. We assume that pain is effectively zero between reports and that the occurrence of pain events exhibits stochastic behavior with temporal dependencies. Even though a lack of report on a particular day is assumed equivalent to a non-event, we acknowledge this assumption may introduce some error given potential reporting fatigue or missed entries.

#### Model fit and distinction.

**Fig 7:**
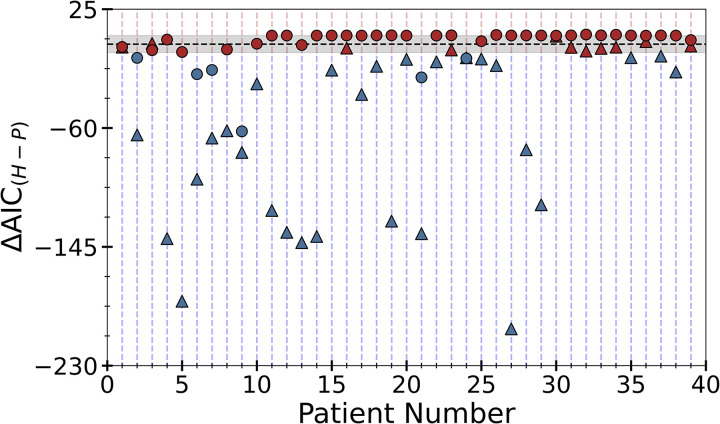
Augmentation method applied to patient data. We plot the difference in AIC (Hawkes minus Poisson) for each time series before (circles) and after (triangles) augmentation. Grey band corresponds to the inconclusive zone (red markers), the region below the band indicates a preference for the Hawkes model with at least 95% confidence (blue markers). See the right panel of Fig 3 for the KS-based similarity matrix used in augmentation.

In Fig 7, we show the results of using our data augmentation method to compare Hawkes and Poisson (null) models for real-world datasets from the SCD patient cohort. We illustrate the model comparison via ΔAIC both without (circles) and with (triangles) data augmentation. Color indicates confidence: **red** for inconclusive regions where ΔAIC lies between -6 and 6, and **blue** for Hawkes fits with more than 95% confidence (ΔAIC <−6). Note that the maximum ΔAIC is + 6 since the Hawkes model reduces to the Poisson model with appropriate parameter choice, and there is a difference of three in the number of degrees of freedom.

While the single-series fits exhibit a preponderance of cases with preference for the Poisson null model over the Hawkes model (28 of 39), the augmented fits demonstrate a preference for the Hawkes model in an overwhelming majority of cases (36 of 39)—see Table 2. Notably, Hawkes model selection for augmented datasets occurred even in many cases where the non-augmented dataset had ΔAIC ≈ 6, the strongest case for the Poisson null model.

**Table 2:** Comparison of AIC-based model preferences: single series fit vs augmented fit. “Basic Preference” indicates the model with the lower AIC. Confidence level 0.05 reflects a strong preference for one model (|ΔAIC|≳6), and confidence level 0.01 reflects a very strong preference for one model (|ΔAIC|≳9.2).

Series Type	Confidence Level	Corresponding |ΔAIC|	# Poisson Patients	# Hawkes Patients
Single	Basic Preference	0	28	11
	0.05	6	2	6
	0.01	9.2	0	6
Augmented	Basic Preference	0	3	37
	0.05	0	0	29
	0.01	9.2	0	27

These results provide compelling evidence for the advantage of leveraging collective likelihoods based on patient similarity in enhancing both model selection and parameter estimation. A negative control experiment confirming that this improvement requires genuine shared structure among pooled series is provided in S4 Appendix. The observed increase in preference for the Hawkes model aligns well with the hypothesis that temporal dependencies play a crucial role in the data.

## Discussion

We have introduced a data augmentation strategy that leverages statistical similarity among sparse time series to improve model selection and parameter estimation—a challenge arising across many scientific domains where individual observations are limited but temporal dependencies are expected.

### Methodological contributions

The augmentation approach addresses two key challenges in analyzing sparse event data. First, it enables model discrimination when individual time series lack sufficient events for conclusive selection. Second, it provides robust parameter estimates by treating statistically similar datasets as multiple realizations of the same underlying process. The collective likelihood framework (Eq. 5) is general and applicable beyond point processes or any specific domain.

Our augmentation approach improves precision by increasing the effective sample size while preserving temporal structure, without requiring assumptions about the specific form of dependencies.

### Key assumption

For the augmentation method to be applicable, there must be a reasonable expectation that the pooled datasets share a common underlying generative process, that is, they reflect the same physical or biological phenomenon. This is not a limitation on the method’s validity, but rather the theoretical basis on which it operates: pooling datasets that truly arise from the same process allows them to be treated as independent realizations of that process, which is precisely what makes the collective likelihood meaningful. When this condition is satisfied, the method yields reliable inference; when it is violated — for instance, when time series arise from experiments governed by vastly different dynamics or entirely different equations — the method should not be applied, as it would produce erroneous and misleading statistical support. S4 Appendix provides empirical characterization of the conditions under which this requirement is satisfied.

In the case of SCD, experts in the field typically classify patients into a limited number of categories [43,44], lending weight to the convenient modeling assumption that they can be clustered based on dynamics.

### Limitations

The KS-based similarity assessment may not capture all temporal structure. Non-transitivity (Fig 3) allows augmented datasets to differ across units but introduces potential selection bias. More sophisticated clustering incorporating domain-specific covariates could refine grouping [16,17].

Different Hawkes parameters can produce similar interarrival distributions if branching ratios match (S2 Appendix). External covariates could help resolve this degeneracy. The exponential kernel assumes a single memory timescale; many processes involve multiple scales. Constant baseline intensity ignores periodic patterns or trends. Extensions incorporating time-varying parameters, compound kernels, or covariates could address these while preserving the core strategy [45,46].

Computational costs scale as *O*(*m*^2^) for similarity testing and *O*(*mk*) for optimization, where *m* is the number of units and *k* is average group size. For large datasets, approximate methods or hierarchical clustering could improve scalability.

### Comparison with existing approaches

Traditional approaches to sparse data include bootstrapping [18] and space-filling algorithms [19], which generate synthetic observations, and Bayesian methods [16], which incorporate prior information. Our approach differs fundamentally: rather than augmenting individual datasets with synthetic or prior-based data, we pool real observations across statistically similar units. This preserves the empirical nature of inference while increasing effective sample size.

Hierarchical models [17,47] also pool information across related groups but require explicit nested structure and shared parameter assumptions. Our similarity-based approach is more flexible, allowing non-hierarchical grouping based on empirical distributional properties. The non-transitivity of similarity (Fig 3) means each unit can be augmented with a different subset of the collection, enabling heterogeneous pooling not possible in standard hierarchical frameworks.

For point processes specifically, most augmentation strategies focus on spatial pooling or assume homogeneity across units [45]. Our approach shares conceptual similarities with Kriging-based methods in spatial statistics, which also pool information across related observations using kernel-based covariance structures [20]. However, while Kriging and its spatiotemporal extensions [21] operate on continuously observed fields with explicit spatial or temporal covariance models, our method is designed for discrete event sequences with no assumed parametric similarity structure—similarity is assessed empirically via a nonparametric test on observed data. Our framework—showcased using Hawkes process—instead performs likelihood-based inference on discrete event times without assuming an underlying continuous trajectory, reflecting our modeling assumption of no latent activity between observed reports. Our temporal similarity assessment via interarrival distributions provides a principled criterion for identifying poolable units without requiring spatial structure or homogeneity assumptions.

### Application to SCD pain dynamics

Our application to sickle cell disease pain events demonstrates practical utility in a real-world clinical context. The shift from 15% to 74% of patients showing confident support for a self-exciting process model has implications for management. It suggests, e.g., that treatment could be improved by enhanced monitoring during high-risk periods following acute episodes, with duration dictated by the memory timescale $\left(\delta^{-1}\right)$ (which we found to range from 30 seconds to 6 minutes in our data—suggesting risk should return to baseline within about 30 minutes). The inferred memory timescales likely reflect rapid physiological fluctuations, such as inflammatory responses or blood pressure dynamics, that operate on minute-to-hour timescales. Though the data collection strategy was set up to avoid missing data, in the plausible case of reporting fatigue, sensitivity of inferred dynamics to missing data is assessed in S4 Appendix.

Current guidelines emphasize reactive treatment [40,41], but temporal dependencies suggest that interventions preventing initial events or breaking excitation cycles during vulnerable periods may be more effective. The branching ratio α/δ quantifies self-excitation strength—patients with higher ratios may benefit from aggressive early intervention to prevent cascades, enabling personalized protocols based on individual temporal dynamics [8,39].

### Future directions

Immediate next steps include validation on independent SCD cohorts to assess generalizability, and application to other temporal event datasets where ground truth is known (e.g., simulated epidemic data with known self-exciting parameters). For SCD, integration of clinical covariates (hemoglobin levels, genotype, treatment) into the similarity assessment may improve patient grouping beyond interarrival times alone.

Extensions to marked point processes (see, e.g., [48] or [49]) could incorporate event severity, addressing a key limitation in the SCD application where pain intensity varies. Time-varying Hawkes models [46] combined with our strategy could capture transitions between acute and chronic pain states [50,51]. Each extension maintains the core principle: leveraging similarity to overcome individual data sparsity.

Deep learning approaches have recently tackled related challenges in other domains: graph contrastive methods have been applied to identify latent structure from sparse biological sequences in an interpretable, consensus-driven manner [52], while debiasing frameworks have been developed to ensure that model selection reflects true generative structure rather than spurious correlations [53]. Adapting such architectures to temporal point process settings could offer complementary advantages in data-rich regimes where likelihood-based methods are limited by model assumptions.

In the work we present here we have manually selected appropriate dataset augmentation thresholds $p_{c}$ for each numerical experiment. Though clearly of interest, we defer for future work the challenge of automatically determining a reasonable threshold, which is connected to the problem of clustering / community detection on a weighted network (the analogue of our p-value matrix **P**).

Finally, we have presented our method in the context of selecting among two point process models, but we believe it could be adapted for selection among more than two candidate models and also for continuous time mechanistic models (e.g., dynamical systems), though AIC may need to be employed with caution in such cases [30].

## Conclusions

This study introduces a data augmentation strategy for temporal event modeling that addresses challenges posed by sparse individual time series. By pooling statistically similar datasets through collective likelihoods, the approach enables reliable model selection and robust parameter estimation when individual units contain insufficient events for conclusive inference.

We demonstrate the use of this method in the context of pain event data for a collection of 39 patients with sickle cell disease. The method’s applicability ultimately rests on a key assumption that multiple sparse datasets originated from the same (or nearly the same) model. In situations where this is plausible, we expect our framework to enable reliable inference from fragmented data, advancing our ability to understand and predict the dynamics of complex systems.

## Supporting information

S1 Appendix Equilibrium intensity for stationary Hawkes process. Brief derivation of expected value formula for Hawkes process intensity.
(PDF)

S2 AppendixIdentifiability of (α,δ) in the exponential Hawkes process.Analysis of practical non-identifiability and likelihood surface properties.(PDF)

S3 Appendix Small-sample correction for model selection. Comparison using corrected AIC (AICc) showing similar results to standard AIC.
(PDF)

S4 Appendix Properties and limitations of the KS-based similarity criterion. Systematic analyses of the augmentation pipeline dependence on our chosen similarity test.
(PDF)

S1 Data Dataset. De-identified dataset containing patient-reported pain scores. (MAT)

S1 Code Python script to load the dataset. (PY)

## References

[1] Pyper E, McKeown S, Hartmann-Boyce J, Powell J. Digital Health Technology for Real-World Clinical Outcome Measurement Using Patient-Generated Data: Systematic Scoping Review. J Med Internet Res. 2023;25:e46992. doi: 10.2196/46992 37819698 PMC10600647

[2] Nowell WB, Curtis JR, Zhao H, Xie F, Stradford L, Curtis D, et al. Participant Engagement and Adherence to Providing Smartwatch and Patient-Reported Outcome Data: Digital Tracking of Rheumatoid Arthritis Longitudinally (DIGITAL) Real-World Study. JMIR Hum Factors. 2023;10:e44034. doi: 10.2196/44034 37934559 PMC10664008

[3] Kang HS, Exworthy M. Wearing the Future-Wearables to Empower Users to Take Greater Responsibility for Their Health and Care: Scoping Review. JMIR Mhealth Uhealth. 2022;10(7):e35684. doi: 10.2196/35684 35830222 PMC9330198

[4] Wettstein R, Sedaghat-Hamedani F, Heinze O, Amr A, Reich C, Betz T, et al. A Remote Patient Monitoring System With Feedback Mechanisms Using a Smartwatch: Concept, Implementation, and Evaluation Based on the activeDCM Randomized Controlled Trial. JMIR Mhealth Uhealth. 2024;12:e58441. doi: 10.2196/58441 39365164 PMC11624455

[5] Gagnon M-P, Ouellet S, Attisso E, Supper W, Amil S, Rhéaume C, et al. Wearable Devices for Supporting Chronic Disease Self-Management: Scoping Review. Interact J Med Res. 2024;13:e55925. doi: 10.2196/55925 39652850 PMC11667132

[6] Iovanel G, Ayers D, Zheng H. The Role of Wearable Technology in Measuring and Supporting Patient Outcomes Following Total Joint Replacement: Review of the Literature. JMIR Perioper Med. 2023;6:e39396. doi: 10.2196/39396 36633891 PMC9880809

[7] Hassan L, Milton A, Sawyer C, Casson AJ, Torous J, Davies A, et al. Utility of Consumer-Grade Wearable Devices for Inferring Physical and Mental Health Outcomes in Severe Mental Illness: Systematic Review. JMIR Ment Health. 2025;12:e65143. doi: 10.2196/65143 39773905 PMC11751658

[8] Vuong C, Utkarsh K, Stojancic R, Subramaniam A, Fernandez O, Banerjee T, et al. Use of consumer wearables to monitor and predict pain in patients with sickle cell disease. Front Digit Health. 2023;5:1285207. doi: 10.3389/fdgth.2023.1285207 37954032 PMC10634543

[9] Stojancic RS, Subramaniam A, Vuong C, Utkarsh K, Golbasi N, Fernandez O, et al. Predicting Pain in People With Sickle Cell Disease in the Day Hospital Using the Commercial Wearable Apple Watch: Feasibility Study. JMIR Form Res. 2023;7:e45355. doi: 10.2196/45355 36917171 PMC10131899

[10] Teng X, Pei S, Lin Y-R. StoCast: Stochastic Disease Forecasting With Progression Uncertainty. IEEE J Biomed Health Inform. 2021;25(3):850–61. doi: 10.1109/JBHI.2020.3006719 32750951

[11] Kaplan AD, Tipnis U, Beckham JC, Kimbrel NA, Oslin DW, McMahon BH, et al. Continuous-time probabilistic models for longitudinal electronic health records. J Biomed Inform. 2022;130:104084. doi: 10.1016/j.jbi.2022.104084 35533991

[12] Liu J, Spakowicz DJ, Ash GI, Hoyd R, Ahluwalia R, Zhang A, et al. Bayesian structural time series for biomedical sensor data: A flexible modeling framework for evaluating interventions. PLoS Comput Biol. 2021;17(8):e1009303. doi: 10.1371/journal.pcbi.1009303 34424894 PMC8412351

[13] Babyak MA. What you see may not be what you get: a brief, nontechnical introduction to overfitting in regression-type models. Psychosomatic Medicine. 2004;66(3):411–21. doi: 10.1097/01.psy.0000127692.23278.a9 15184705

[14] Ioannidis JPA. Why most published research findings are false. PLoS Med. 2005;2(8):e124. doi: 10.1371/journal.pmed.0020124 16060722 PMC1182327

[15] Riley RD, Snell KI, Ensor J, Burke DL, Harrell FE Jr, Moons KG, et al. Minimum sample size for developing a multivariable prediction model: PART II - binary and time-to-event outcomes. Stat Med. 2019;38(7):1276–96. doi: 10.1002/sim.7992 30357870 PMC6519266

[16] Gelman A, Carlin JB, Stern HS, Dunson DB, Vehtari A, Rubin DB. Bayesian Data Analysis. 3rd ed. Chapman & Hall/CRC. 2013.

[17] Raudenbush SW. Hierarchical linear models: Applications and data analysis methods. Advanced Quantitative Techniques in the Social Sciences Series: SAGE. 2002.

[18] Efron B, Tibshirani RJ. An Introduction to the Bootstrap. Chapman & Hall/CRC. 1994.

[19] McKay MD, Beckman RJ, Conover WJ. Comparison of Three Methods for Selecting Values of Input Variables in the Analysis of Output from a Computer Code. Technometrics. 1979;21(2):239–45. doi: 10.1080/00401706.1979.10489755

[20] Cressie N. Statistics for spatial data. John Wiley & Sons. 2015.

[21] Wikle CK, Zammit-Mangion A, Cressie N. Spatio-Temporal Statistics with R. Boca Raton: Chapman and Hall/CRC. 2019.

[22] Adesina O, Brunson A, Fisch MJ, Wun T, Shi Q. All-cause 30-day readmission rate and risk factors in patients with sickle cell disease: A population-based cohort study. American Journal of Hematology. 2023;98(5):730–8. doi: 10.1002/ajh.2687236869876

[23] Shah N, Bhor M, Xie L, Paulose J, Yuce H. Sickle cell disease complications: Prevalence and resource utilization. PLoS One. 2019;14(7):e0214355. doi: 10.1371/journal.pone.0214355 31276525 PMC6611562

[24] Hawkes AG. Spectra of some self-exciting and mutually exciting point processes. Biometrika. 1971;58(1):83–90. doi: 10.1093/biomet/58.1.83

[25] Hawkes AG, Oakes D. A cluster process representation of a self-exciting process. Journal of Applied Probability. 1974;11(3):493–503. doi: 10.2307/3212693

[26] Ogata Y. Statistical Models for Earthquake Occurrences and Residual Analysis for Point Processes. Journal of the American Statistical Association. 1988;83(401):9–27. doi: 10.1080/01621459.1988.10478560

[27] Mohler GO, Short MB, Brantingham PJ, Schoenberg FP, Tita GE. Self-Exciting Point Process Modeling of Crime. Journal of the American Statistical Association. 2011;106(493):100–8. doi: 10.1198/jasa.2011.ap09546

[28] Bowsher CG. Modelling security market events in continuous time: Intensity based, multivariate point process models. Journal of Econometrics. 2007;141(2):876–912. doi: 10.1016/j.jeconom.2006.11.007

[29] Akaike H. A new look at the statistical model identification. IEEE Trans Automat Contr. 1974;19(6):716–23. doi: 10.1109/tac.1974.1100705

[30] Utkarsh K, Abrams DM. Information criteria fail for dynamical systems: sampling rate and dimension dependence. arXiv. 2025. 2511.14931.

[31] Hurvich CM, Tsai C-L. Regression and time series model selection in small samples. Biometrika. 1989;76(2):297–307. doi: 10.1093/biomet/76.2.297

[32] Hurvich CM, Tsai C-L. Regression and time series model selection in small samples. Biometrika. 1989;76(2):297–307. doi: 10.1093/biomet/76.2.297

[33] Piel FB, Patil AP, Howes RE, Nyangiri OA, Gething PW, Dewi M, et al. Global epidemiology of sickle haemoglobin in neonates: a contemporary geostatistical model-based map and population estimates. Lancet. 2013;381(9861):142–51. doi: 10.1016/S0140-6736(12)61229-X 23103089 PMC3547249

[34] Hassell KL. Population estimates of sickle cell disease in the U.S. Am J Prev Med. 2010;38(4 Suppl):S512-21. doi: 10.1016/j.amepre.2009.12.022 20331952

[35] Platt OS, Thorington BD, Brambilla DJ, Milner PF, Rosse WF, Vichinsky E, et al. Pain in sickle cell disease. Rates and risk factors. N Engl J Med. 1991;325(1):11–6. doi: 10.1056/NEJM199107043250103 1710777

[36] Shah N, Jonassaint J, De Castro L. Patients welcome the Sickle Cell Disease Mobile Application to Record Symptoms via Technology (SMART). Hemoglobin. 2014;38(2):99–103. doi: 10.3109/03630269.2014.880716 24512633

[37] Shah N, Jonassaint J, De Castro L. Patients welcome the Sickle Cell Disease Mobile Application to Record Symptoms via Technology (SMART). Hemoglobin. 2014;38(2):99–103. doi: 10.3109/03630269.2014.880716 24512633

[38] Shah N, Jonassaint J, De Castro L. A digital health intervention for acute pain management in sickle cell disease: pilot study. JMIR mHealth and uHealth. 2019;7(4):e11791. doi: 10.2196/11791 PMC6915456 31789599

[39] Smith WR, Penberthy LT, Bovbjerg VE, McClish DK, Roberts JD, Dahman B, et al. Daily assessment of pain in adults with sickle cell disease. Ann Intern Med. 2008;148(2):94–101. doi: 10.7326/0003-4819-148-2-200801150-00004 18195334

[40] National Academies of Sciences, Engineering, and Medicine. Addressing Sickle Cell Disease: A Strategic Plan and Blueprint for Action. Washington, DC: The National Academies Press. 2020. 33411430

[41] National Academies of Sciences, Engineering, and Medicine. Sickle Cell Disease in Social Security Disability Evaluations: Pain and Treatment Settings. Washington, DC: The National Academies Press. 2025. 41284795

[42] Stewart KA, Parshad-Asnani M, Wonkam A, Bollinger J, Ngo Bitoungui V, Wonkam-Tingang E, et al. “Pain is Subjective”: A Mixed-Methods Study of Provider Attitudes and Practices Regarding Pain Management in Sickle Cell Disease Across Three Countries. J Pain Symptom Manage. 2021;61(3):474–87. doi: 10.1016/j.jpainsymman.2020.08.029 32889040

[43] Shah N, Beenhouwer D, Broder MS, Bronte-Hall L, De Castro LM, Gibbs SN, et al. Development of a Severity Classification System for Sickle Cell Disease. Clinicoecon Outcomes Res. 2020;12:625–33. doi: 10.2147/CEOR.S276121 33149635 PMC7604906

[44] Ballas SK, Lieff S, Benjamin LJ, Dampier CD, Heeney MM, Hoppe C, et al. Definitions of the phenotypic manifestations of sickle cell disease. Am J Hematol. 2010;85(1):6–13. doi: 10.1002/ajh.21550 19902523 PMC5046828

[45] Reinhart A. A Review of Self-Exciting Spatio-Temporal Point Processes and Their Applications. Statist Sci. 2018;33(3). doi: 10.1214/17-sts629

[46] Chiang W-H, Liu X, Mohler G. Hawkes process modeling of COVID-19 with mobility leading indicators and spatial covariates. Int J Forecast. 2022;38(2):505–20. doi: 10.1016/j.ijforecast.2021.07.001 34276115 PMC8275517

[47] Goldstein H. Multilevel statistical models. John Wiley & Sons. 2011.

[48] Schlather M. On the Second-Order Characteristics of Marked Point Processes. Bernoulli. 2001;7(1):99. doi: 10.2307/3318604

[49] Lotwick HW, Silverman BW. Methods for Analysing Spatial Processes of Several Types of Points. Journal of the Royal Statistical Society Series B: Statistical Methodology. 1982;44(3):406–13. doi: 10.1111/j.2517-6161.1982.tb01221.x

[50] Carroll CP, Lanzkron S, Haywood CJ, Kiley K, Pejsa M, Moscou-Jackson G. Detecting the emergence of chronic pain in sickle cell disease. Journal of Pain and Symptom Management. 2018;55(4):1131–9. doi: 10.1016/j.jpainsymman.2017.12.48329221847

[51] Bakshi N, Smith ME, Ross D, Krishnamurti L. Novel Metrics in the Longitudinal Evaluation of Pain Data in Sickle Cell Disease. Clin J Pain. 2017;33(6):517–27. doi: 10.1097/AJP.0000000000000431 27584817

[52] Li G, Zhao B, Su X, Yang Y, Hu P, Zhou X, et al. Discovering Consensus Regions for Interpretable Identification of RNA N6-Methyladenosine Modification Sites via Graph Contrastive Clustering. IEEE J Biomed Health Inform. 2024;28(4):2362–72. doi: 10.1109/JBHI.2024.3357979 38265898

[53] Zeng Z, Luo M, Kong X, Liu H, Guo H, Yang H, et al. Mitigating World Biases: A Multimodal Multi-View Debiasing Framework for Fake News Video Detection. In: Proceedings of the 32nd ACM International Conference on Multimedia, 2024. 6492–500. 10.1145/3664647.3681673

