# Supplementary material for: A new method for augmenting short time series, with application to pain events in sickle cell disease
Source: PLoS Comput Biol. 2026 Jun 12;22(6):e1014389. doi: 10.1371/journal.pcbi.1014389 (PMC13286270; doi:10.1371/journal.pcbi.1014389)
Supplement: S1 Appendix — Brief derivation of expected value formula for Hawkes process intensity. (PDF) [file pcbi.1014389.s001.pdf]

S1 Appendix: Stationary Mean Intensity of a Hawkes Process

Kumar Utkarsh, Nirmish R. Shah, Tanvi Banerjee, Daniel M. Abrams

Consider a Hawkes process with baseline intensity λ_0 and an exponential kernel

$$\Phi(t) = \alpha e^{-\delta t}, \quad t \geq 0.$$

The conditional intensity at time t is given by

$$\lambda(t) = \lambda_0 + \sum_{i:t_i<t} \Phi(t - t_i) = \lambda_0 + \alpha \sum_{i:t_i<t} e^{-\delta(t-t_i)},$$

where the sum is over all events that occurred before time t. Each past event contributes to the current intensity, with a contribution that decays exponentially over time.

Branching process interpretation. One way to look at the Hawkes process is through its branching structure. Each event can be thought of [1] as generating future "offspring" events independently according to a Poisson process with mean

$$\eta = \int_0^\infty \Phi(t)\, dt = \frac{\alpha}{\delta}.$$

This quantity η is called the *branching ratio*. If $\eta < 1$, the process is subcritical, meaning that on average each event produces less than one descendant. This ensures that the process does not explode and admits a stationary distribution.

Stationary mean intensity. Let $\lambda_c = \mathbb{E}[\lambda(t)]$ denote the stationary mean intensity. By stationarity, the expected total intensity can be decomposed into the baseline intensity plus the expected contribution from all offspring events. In other words, each event contributes to future intensity on average according to the branching ratio:

$$\lambda_c = \lambda_0 + \eta\, \lambda_c.$$

Here the first term λ_0 accounts for the spontaneous events, while the second term $\eta\, \lambda_c$ accounts for the self-excitation of the process in expectation. Solving for λ_c gives the exact stationary mean intensity:

$$\lambda_c = \frac{\lambda_0}{1 - \eta} = \frac{\lambda_0}{1 - \alpha/\delta}. \tag{1}$$

The branching ratio η provides an intuitive measure of how strongly the process self-excites: as $\eta \to 1$, the stationary mean diverges, reflecting the transition to a critical process.

References

[1] Laub PJ, Lee Y, Taimre T. The Elements of Hawkes Processes. Springer; 2021.
