# Supplementary material for: A new method for augmenting short time series, with application to pain events in sickle cell disease
Source: PLoS Comput Biol. 2026 Jun 12;22(6):e1014389. doi: 10.1371/journal.pcbi.1014389 (PMC13286270; doi:10.1371/journal.pcbi.1014389)
Supplement: S2 Appendix — Analysis of practical non-identifiability and likelihood surface properties. (PDF) [file pcbi.1014389.s002.pdf]

S2 Appendix: Identifiability of (α, δ) in the Exponential Hawkes Process

Kumar Utkarsh, Nirmish R. Shah, Tanvi Banerjee, Daniel M. Abrams

In Fig 5 of the main text, we note that the estimated parameters converge nearly—but not exactly—to the true values; this is a result of a near-failure of parameter identifiability for the Hawkes model. True failure would occur if one or more likelihood contours in the (α, δ)-plane became straight lines (see Fig 1).

The issue is not that the model is theoretically unidentifiable, but that in finite samples (especially with short observation windows or few events) the likelihood surface becomes nearly constant along certain curves in the (α, δ)-plane, making different parameter pairs yield almost indistinguishable fits.

Consider the Hawkes process with exponential memory kernel

$$\Phi(t) = \alpha e^{-\delta t}, \qquad \alpha > 0,\ \delta > 0, \tag{1}$$

and intensity

$$\lambda(t) = \lambda_0 + \sum_{i:t_i<t} \alpha e^{-\delta(t-t_i)}. \tag{2}$$

For event times $\{t_i\}_{i=1}^N$ on $[0, T]$, the log-likelihood is

$$\mathcal{L}(\alpha, \delta) = \underbrace{\sum_{i=1}^{N} \log \lambda(t_i)}_{\text{log–intensity at events}} - \underbrace{\int_0^T \lambda(t)\, dt}_{\text{compensator}}. \tag{3}$$

For the exponential kernel, the compensator (the integrated intensity function) has the closed form

$$\int_0^T \lambda(t)\, dt = \lambda_0 T + \frac{\alpha}{\delta} \sum_{i=1}^{N} \left(1 - e^{-\delta(T-t_i)}\right). \tag{4}$$

When both the decay rate δ is sufficiently large and events occur sufficiently early in the observation window such that $\delta(T - t_i) \gg 1$ for most events, the terms satisfy $e^{-\delta(T-t_i)} \approx 0$. In this regime,

$$\int_0^T \lambda(t)\, dt \approx \lambda_0 T + \frac{\alpha}{\delta} N, \tag{5}$$

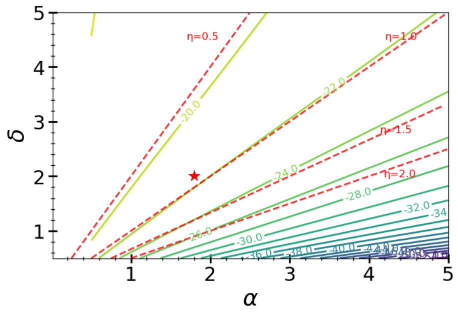

Figure 1: **Likelihood ridge along constant branching ratio reveals practical non-identifiability.** Practical non-identifiability of (α, δ) in the exponential Hawkes process. Log-likelihood surface for synthetic data with $N = 6$ events over $[0, 10]$, showing contours and lines of constant branching ratio $\eta = \alpha/\delta$ (red dashed). The true parameters ($\alpha = 1.8$, $\delta = 2.0$, red star) lie on the $\eta = 0.9$ ridge. Likelihood contours run nearly parallel to constant-η lines, creating a flat direction. The numerical evaluation shows log-likelihood varies by only $\Delta \log L \approx 0.8$ across a wide range of α values for $\eta = 0.9$, demonstrating a relatively weaker constraint on individual parameters despite a strong constraint on their ratio. This flatness explains why MLE optimization often yields parameter estimates that converge close to, but not exactly at, the true values.

so the integral depends almost entirely on the branching ratio $\eta = \alpha/\delta$. This produces an exactly linear contour in the likelihood plane coming from the compensator alone.

The intensity at an event time is

$$\lambda(t_i) = \lambda_0 + \sum_{j<i} \alpha \, e^{-\delta(t_i - t_j)}. \tag{6}$$

The contribution of each past event involves the product $\alpha e^{-\delta \Delta t}$, where $\Delta t = t_i - t_j$. In principle this term distinguishes α and δ, since it does not collapse to a function of α/δ. However, the excitation contributions $\sum_{j<i} \alpha e^{-\delta(t_i - t_j)}$ become negligible compared to λ_0 when either (i) the excitation is weak ($\alpha/\lambda_0 \ll 1$), making the process only weakly self-exciting, or (ii) the decay rate δ is large relative to typical inter-event times, causing excitations from past events to die out before the next event occurs. In these regimes, $\lambda(t_i) \approx \lambda_0$ for most events, so the log-intensity contributions $\log \lambda(t_i)$ vary only weakly with (α, δ). Consequently the event-time term cannot significantly bend the nearly linear contours produced by the compensator.

Combining the two effects:

- the compensator term is nearly invariant along curves of constant α/δ, and

- the event-time intensities only weakly constrain (α, δ) unless the data contain inter-event gaps that probe the exponential decay scale,

the log-likelihood develops a nearly constant ridge along curves close to

$$\{(\alpha, \delta) : \alpha/\delta \approx \text{constant}\}. \tag{7}$$

This explains why parameter estimates often converge close to — but not exactly equal to—the true values unless large datasets or very tight optimization tolerances are available. We can visualize the ridges in Fig 1.
