# Supplementary material for: A new method for augmenting short time series, with application to pain events in sickle cell disease
Source: PLoS Comput Biol. 2026 Jun 12;22(6):e1014389. doi: 10.1371/journal.pcbi.1014389 (PMC13286270; doi:10.1371/journal.pcbi.1014389)
Supplement: S3 Appendix — Comparison using corrected AIC (AICc) showing similar results to standard AIC. (PDF) [file pcbi.1014389.s003.pdf]

S3 Appendix: Small-Sample Correction for Model Selection

Kumar Utkarsh, Nirmish R. Shah, Tanvi Banerjee, Daniel M. Abrams

To assess the impact of small datasets on model selection, we recomputed the model comparison using the corrected Akaike Information Criterion (AICc) [1]. Fig 1 shows results analogous to Fig 2 from the main text, illustrating how AICc performs almost equivalently to AIC qualitatively.

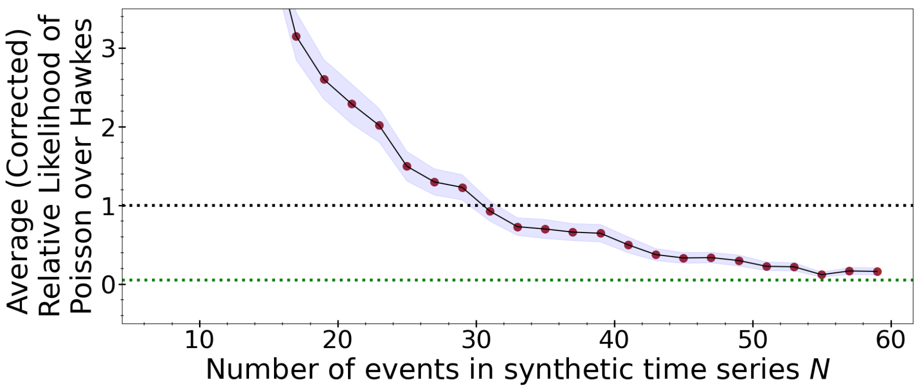

Figure 1: **AICc correction does not resolve sparse-data model selection challenges.** Number of data points needed to distinguish the Hawkes model from the Poisson model using AICc. As in Fig 2 of the main text, black dashed line shows basic preference ($\mathcal{L} = 1$), green dashed line shows 95% confidence ($\mathcal{L} = 0.05$), red markers indicate averages over 50 repetitions, and purple shading denotes the 95% confidence interval. Parameter choices: $(\lambda_0, \alpha, \delta)$=(1, 3, 6).

References

[1] Hurvich CM, Tsai CL. Regression and time series model selection in small samples. Biometrika. 1989;76(2):297–307. doi:10.1093/biomet/76.2.297.
