# Supplementary material for: A new method for augmenting short time series, with application to pain events in sickle cell disease
Source: PLoS Comput Biol. 2026 Jun 12;22(6):e1014389. doi: 10.1371/journal.pcbi.1014389 (PMC13286270; doi:10.1371/journal.pcbi.1014389)
Supplement: S4 Appendix — Systematic analyses of the augmentation pipeline dependence on our chosen similarity test. (PDF) [file pcbi.1014389.s004.pdf]

S4 Appendix: Properties and Limitations of the KS-Based Similarity Criterion

Kumar Utkarsh, Nirmish R. Shah, Tanvi Banerjee, Daniel M. Abrams

The data augmentation method proposed in the main text relies on the two-sample Kolmogorov–Smirnov (KS) test applied to interarrival-time distributions to decide which datasets should be pooled. This appendix characterises the sensitivity and limitations of that criterion through a series of numerical experiments. The central finding is that the criterion is *robust for the model selection task it is asked to perform*, but that the choice of similarity threshold p_c is fundamentally dependent on series length and on which aspects of the distribution differ between series. We also characterise the sensitivity of the method to missing data. Principled, automatic threshold selection is a non-trivial open problem that we defer to future work.

1 KS vs. Anderson–Darling: justification of test choice

We first verify that replacing the KS test with the Anderson–Darling (AD) two-sample test does not yield a materially better similarity criterion. Fig 1 reports precision, recall, and F1 score for both tests at significance level $\alpha = 0.10$, evaluated on $N = 200$ positive pairs (series drawn from identical Hawkes parameters $\lambda_0 = \lambda_0^*, \alpha = \alpha^*, \delta = \delta^*$) and $N = 200$ negative pairs (series drawn from independently sampled parameter sets). The KS test achieves precision 0.69, recall 0.92, and F1 0.79; the AD test achieves precision 0.64, recall 0.86, and F1 0.73. KS performs slightly better on all three metrics at this sample size, and its p-values are analytically exact and computationally cheaper. We therefore retain the KS test throughout.

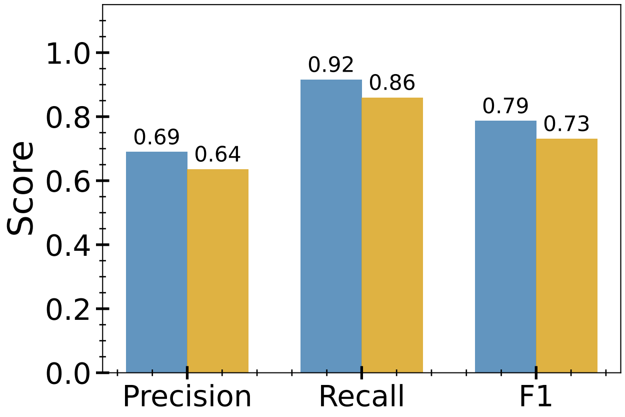

Figure 1: **KS test matches or exceeds Anderson–Darling for similarity classification at realistic data sizes.** Precision (left), recall (center), and F1 score (right) for the two-sample KS test (blue) and Anderson–Darling test (gold) evaluated at significance level $\alpha = 0.10$. Positive pairs: $N = 200$ pairs of series drawn from the same Hawkes parameter set $(\lambda_0^*, \alpha^*, \delta^*) = (1, 2, 3.5)$. Negative pairs: $N = 200$ pairs where the second series is drawn from a randomly sampled parameter set. Each bar is a single-run estimate; stochastic variability across runs is small relative to the gap between the two tests.

2 KS sensitivity to mean rate

The KS test is applied to interarrival-time distributions, whose shape for a Hawkes process is governed primarily by the mean rate $\lambda_c = \lambda_0/(1-\alpha/\delta)$. Fig 2 shows the mean KS p-value (± 1 std band across $N = 500$ pairs, $N_\mathrm{e} = 30$) as a function of the rate ratio for two comparison types. The left panel compares two Hawkes series with mean rates $\lambda_c^{(1)}$ and $\lambda_c^{(2)} = r\,\lambda_c^{(1)}$: the mean p-value falls below $p_c = 0.10$ at a ratio of approximately 2. The right panel compares a Hawkes series (mean rate λ_c^{H}) against a Poisson series (rate $\lambda_\mathrm{P} = r\,\lambda_c^{\mathrm{H}}$): the transition occurs at a ratio of approximately 3, reflecting the additional distributional difference between exponential and non-exponential interarrivals that partially offsets the rate difference. In both cases, series with sufficiently similar mean rates are reliably classified as similar at $p_c = 0.10$.

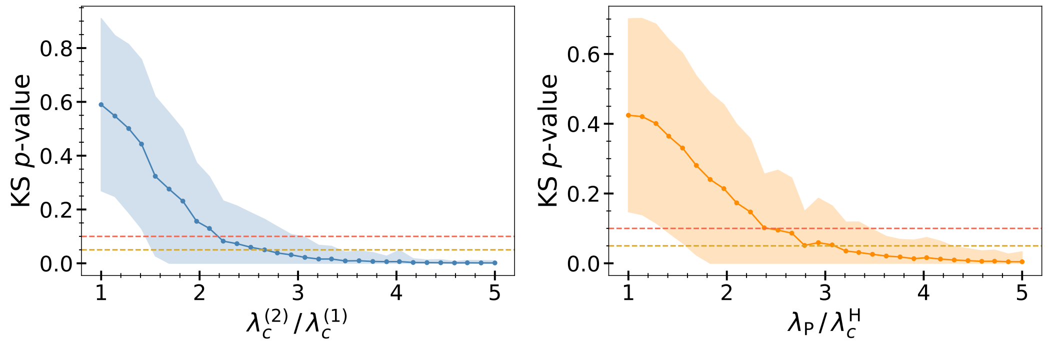

Figure 2: **KS test reliably separates series with sufficiently different mean rates.** Mean KS p-value (± 1 standard deviation across $N_{\mathrm{pairs}} = 500$ pairs, shaded band) as a function of the mean rate ratio, at fixed series length $N_\mathrm{e} = 30$. Red dashed line: $p_c = 0.10$; gold dashed line: $p_c = 0.05$. *Left:* Hawkes vs. Hawkes comparison. Both series are Hawkes processes with the same branching ratio α/δ and decay rate δ; only λ_0 is varied to produce the target ratio $\lambda_c^{(2)}/\lambda_c^{(1)}$. The critical rate parameters is calculated using true parametric values $(\lambda_0^*, \alpha^*, \delta^*) = (1, 2, 3.5)$. The mean p-value falls below $p_c = 0.10$ at a ratio of approximately 2. *Right:* Hawkes vs. Poisson comparison. The Hawkes series uses true parameters $(\lambda_0^*, \alpha^*, \delta^*) = (1, 2, 3.5)$ and the Poisson series has rate $\lambda_\mathrm{P} = r\,\lambda_c^{\mathrm{H}}$. The transition occurs at a ratio of approximately 3, reflecting the additional shape difference between exponential and Hawkes interarrival distributions.

3 Effect of series length on KS discrimination

Fig 3 shows the full p-value distribution for four distributional distance cases at $N_\mathrm{e} = 500$ (top row) and $N_\mathrm{e} = 30$ (bottom row) for two Poisson processes. The two extreme cases—identical distributions (λ ratio $= 1$) and disjoint distributions (λ ratio $= 3$)—are correctly identified at both sample sizes. At $N_\mathrm{e} = 30$ the two intermediate cases (λ ratio 1.25 and 1.75) become difficult to distinguish from the identical case, with matched fractions of 0.88 and 0.56 respectively, compared to 0.13 and 0.00 at $N_\mathrm{e} = 500$. The threshold p_c therefore only matters in this intermediate regime, and its effect is amplified at small N_e.

4 Sensitivity to series length and branching ratio

Fig 4 quantifies two further dimensions of KS sensitivity. The left panel shows that, for a fixed mean rate ratio of 2, the test reliably rejects similarity (mean p-value below $p_c = 0.10$) once series contain approximately 50 events; at $N_\mathrm{e} = 30$ the mean p-value lies close to the threshold, confirming the borderline power noted above. The right panel fixes the mean rate λ_c and varies the branching ratio

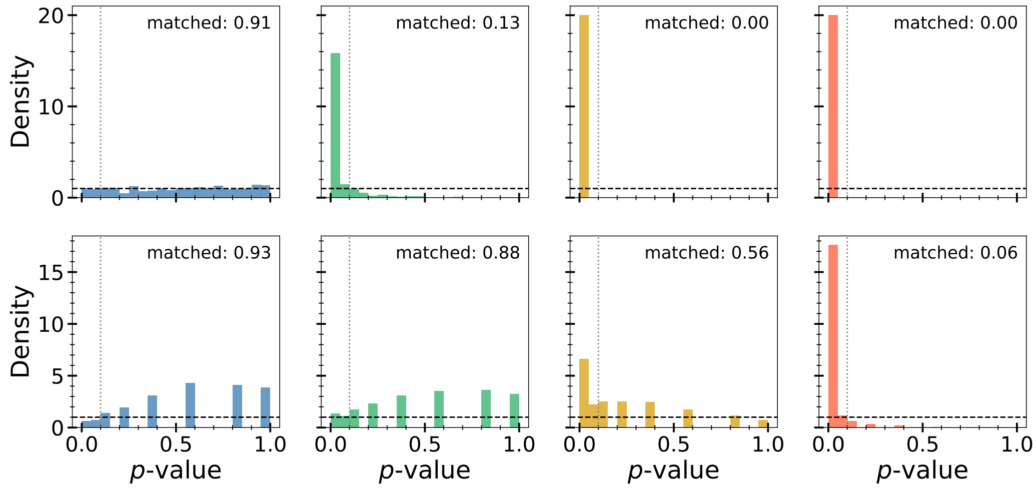

Figure 3: **KS test performs well with abundant data but struggles to distinguish non-identical (but similar) distributions in limited data regime.** Distribution of KS p-values for four Poisson process cases at $N_{\mathrm{e}} = 500$ (top row) and $N_{\mathrm{e}} = 30$ (bottom row, our data regime). Columns from left to right: identical distributions (λ ratio $= 1$), similar (λ ratio $= 1.25$), intermediate (λ ratio $= 1.75$), and disjoint (λ ratio $= 3$). Black dashed horizontal line: uniform density (expected under H_0). Gray dotted vertical line: $p_c = 0.10$. Annotated fraction gives the proportion of $N_{\mathrm{pairs}} = 2000$ pairs matched at $p_c = 0.10$. At $N_{\mathrm{e}} = 30$ the intermediate cases—where the two series being compared have similar but not identical mean rates—produce near-uniform p-value distributions, rendering the threshold choice consequential.

α/δ of the second series while holding the first at $\alpha/\delta = 0.3$ (vertical dashed line). At $N_{\mathrm{e}} = 30$ (green) the p-value is approximately flat across all branching ratios, demonstrating that the KS test on interarrival times is *insensitive to self-excitation structure* at realistic data sizes. At $N_{\mathrm{e}} = 500$ (purple) the test begins to detect differences in branching ratio only when they are large ($|\Delta(\alpha/\delta)| \gtrsim 0.3$). This result implies that the KS similarity criterion groups patients primarily by mean pain event rate, not by the strength of their self-exciting dynamics. Two patients with identical pain frequency but very different excitation structure would be incorrectly pooled. This is a fundamental limitation of the current approach, and resolving it—for instance by incorporating a distance metric that is explicitly sensitive to temporal clustering—constitutes an important future research direction.

5 Leave-one-out stability of parameter estimates

To assess how sensitive the fitted Hawkes parameters are to the exact composition of the augmented group, we perform a leave-one-out (LOO) analysis on a matched collection of $N = 12$ series ($N_{\mathrm{e}} = 30$ events each, $p_c = 0.10$). For each focal series, we identify its KS neighbourhood, fit the Hawkes model to the full neighbourhood, and then refit after removing each neighbour in turn. The coefficient of variation (CV $= \sigma/|\mu|$) across these LOO refits measures how much the parameter estimate changes when a single series is removed. Fig 5 shows the CV for α (blue), δ (red), and the branching ratio α/δ (green) across all focal series for which the neighbourhood contained at least two members. All CV values lie below the reference line at 0.10 (dashed), indicating that no parameter estimate is expected to change by more than 10% when a single series is removed. Mean CVs are approximately 0.045 for α, 0.066 for δ, and 0.046 for α/δ. The decay rate δ shows the greatest sensitivity, consistent with the known flatness of the likelihood surface in the α–δ direction noted in S2 Appendix. The branching ratio α/δ, which governs the overall self-excitation strength, is the most stable of the three. These results confirm that the augmented parameter estimates are not very sensitive to group

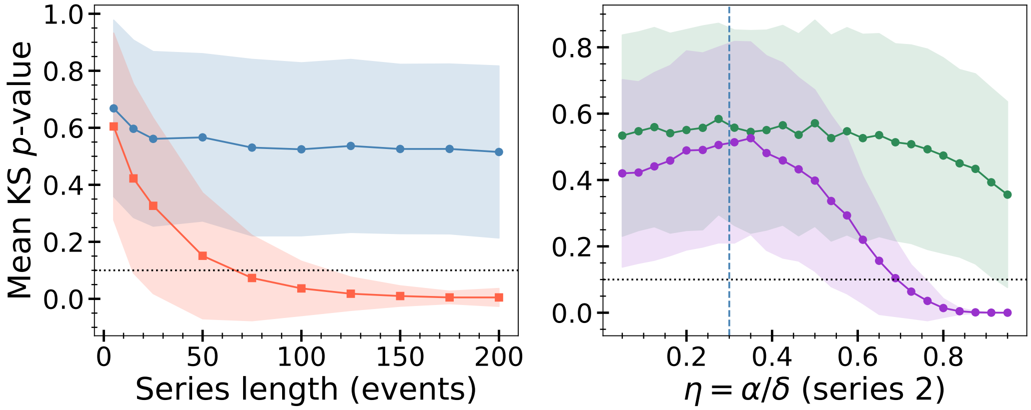

Figure 4: **KS test dependence on series length and branching ratio differences.** *Left:* Mean KS p-value (± 1 std across $N_{\text{pairs}} = 500$ pairs) as a function of series length for pairs with the same mean rate (blue circles, $\lambda_1^* = \lambda_2^* = 1.63$) and pairs with mean rate ratio 2.0 (red squares, $\lambda_1^* = \lambda_2^*/2 = 1.63$). In both cases, $\alpha^* = 0.9$ and $\delta^* = 3$. Dotted horizontal line: $p_c = 0.10$. Both series share branching ratio $\alpha/\delta = 0.3$ and decay rate $\delta = 3$. *Right:* Mean KS p-value (± 1 std) as a function of the branching ratio $\eta = \alpha/\delta$ of the second series, with the first series fixed at $\eta = 0.3$ (vertical dashed blue line). For series 1, $(\lambda_1^*, \alpha_1^*, \delta_1^*) = (1.63, 0.9, 3)$. For series 2, the parametric values are dependent on the $x-$axis ($\eta = \alpha/\delta$), that is $(\lambda_2^*, \alpha_2^*, \delta_2^*) = (1.63(1-\eta), 3\eta, 3)$. Green: $N_{\text{e}} = 30$; purple: $N_{\text{e}} = 500$. The mean rate λ_c is held constant for both series by adjusting λ_0 accordingly. Dotted horizontal line: $p_c = 0.10$. At $N_{\text{e}} = 30$ the curve is approximately flat, showing that the KS criterion cannot distinguish different self-excitation strengths at realistic SCD data sizes (at least for the chosen parameters).

composition at $p_c = 0.10$.

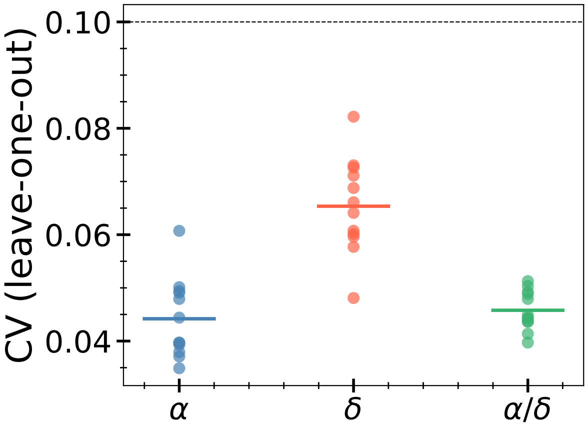

Figure 5: **Parameter estimates are stable under single-member removal from the augmented group.** Coefficient of variation (CV $= \sigma/|\mu|$) of Hawkes parameter estimates across leave-one-out refits, for α (blue), δ (red), and branching ratio α/δ (green). Each dot corresponds to one focal series; horizontal bars show the mean CV across focal series. Dashed line: CV $= 0.10$ reference. Collection of $N = 12$ matched Hawkes series, $N_{\text{e}} = 30$ events per series, $p_c = 0.10$, true parameters $(\lambda_0^*, \alpha^*, \delta^*) = (1, 2, 3.5)$. All CVs fall below 0.10, with δ showing the largest spread (mean CV ≈ 0.07) consistent with the known flatness of the likelihood surface in the α–δ direction.

6 Robustness of model selection to threshold choice

Despite the difficulties in principled p_c selection documented above, we show here that the *model selection conclusion* of the main text is robust to the matching criterion. Fig 6 shows $\Delta\text{AIC} = \text{AIC(Hawkes)} - \text{AIC(Poisson)}$ for three conditions at $p_c = 0.10$: a single unaided Hawkes process time series (blue circle), augmentation of $N_{\text{aug}} = 10$ series drawn from identical parameters (green square, positive control), and augmentation of $N_{\text{aug}} = 10$ series drawn from deliberately dissimilar parameter sets whose mean rates are the same (red diamond, negative control). The single series and related (but dissimilar) augmentation both fall within the inconclusive band ($|\Delta\text{AIC}| < 6$, gray shading), while matched augmentation produces $\Delta\text{AIC} \approx -23$, well beyond the 95% confidence threshold (purple dotted line, $\Delta\text{AIC} = -6$). This demonstrates that the improvement in model selection is not an artifact of pooling per se, but requires genuine shared structure among the pooled series.

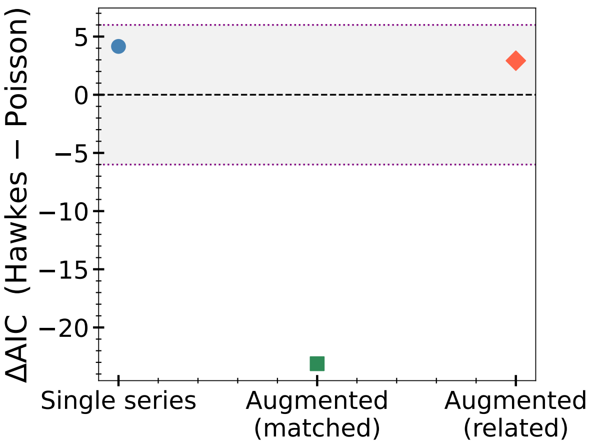

Figure 6: **Augmentation improves model selection only when pooled series share a common generative process.** $\Delta\text{AIC} = \text{AIC(Hawkes)} - \text{AIC(Poisson)}$ for three conditions: single series with no augmentation (blue circle); augmentation of $N_{\text{aug}} = 10$ matched series drawn from the same Hawkes parameters $(\lambda_0^*, \alpha^*, \delta^*) = (1, 2, 3.5)$ (green square, positive control); augmentation of $N_{\text{aug}} = 10$ series drawn from related but dissimilar Hawkes processes. That is, different parametric values but the same mean rates λ_c (red diamond, negative control). To calculate the parameters, we randomly sampled α and δ uniformly from distributions $\mathcal{U}(0.1, 1.5)$ and $\mathcal{U}(\alpha, 4)$ respectively, and calculated λ_0 using the known mean rate. Gray band: inconclusive region $|\Delta\text{AIC}| < 6$. Black dashed line: $\Delta\text{AIC} = 0$. Purple dotted lines: 95% confidence threshold $|\Delta\text{AIC}| = 6$. Similarity threshold $p_c = 0.10$; $N_{\text{e}} = 30$ per series.

False positive rate under the Poisson null

To numerically quantify the risk of incorrectly preferring the Hawkes model when data are truly Poisson, we generate 100 collections of $N_{\text{aug}} = 10$ Poisson series ($N_{\text{e}} = 30$ events each), apply the full augmentation pipeline at $p_c = 0.10$, and record the fraction of series for which ΔAIC falls below the Hawkes-preference threshold. Results are shown in Table 1. Single-series false positive rates (FPRs) are almost negligible. Augmentation raises the tight FPR to 0.098, because pooling increases statistical power indiscriminately when the KS test cannot distinguish Poisson from Hawkes interarrival distributions at $N_{\text{e}} = 30$ (Sec. 4).[1] The method should therefore be used in settings where self-exciting dynamics are plausible a priori, not as a conservative test of the Poisson null.

[1] We note, however, that the maximum likelihood parameters determined in the false positive cases are typically nearly degenerate to the Poisson process: e.g, $\alpha \approx 0$ or $\delta \to \infty$.

Table 1: **False positive rates for truly Poisson data.** FPR across $N_{\text{aug}} = 10$ series with $N_{\text{e}} = 30$.

**Condition**	**Tight (ΔAIC < -6)**	**Loose (ΔAIC < 0)**
Single series	0.001	0.021
Augmented	0.098	0.290

7 Sensitivity to missing data and reporting gaps

In real-world deployment, patient-reported data inevitably contains missing entries due to reporting fatigue or non-compliance. We assess how the method degrades as the fraction of missing events increases. Starting from a single long Hawkes series ($N_{\text{e}} = 300$ events, true parameters $\lambda_0^*, \alpha^*, \delta^*$), we randomly remove a fraction q of events and refit both models to the thinned series. Fig 7 shows the results as a function of q for a more bursty parameter set $(\lambda_0^*, \alpha^*, \delta^*) = (1, 3, 7)$. The left panel shows ΔAIC: the Hawkes model is confidently preferred ($\Delta\text{AIC} < -6$) up to approximately $q = 60\%$ missing data, above which the series becomes too sparse for reliable model discrimination and results become inconclusive or reverse. The right panel shows the relative parameter estimation error $|\hat{\theta} - \theta^*|/\theta^*$ for α (red circles), δ (gold squares), and λ_0 (green triangles). Errors remain reasonable for $q \lesssim 60\%$ and continue growing steeply as q grows. The λ_0 estimate is most robust, while α degrades earliest, consistent with its role in capturing the self-exciting structure that is most sensitive to event removal.

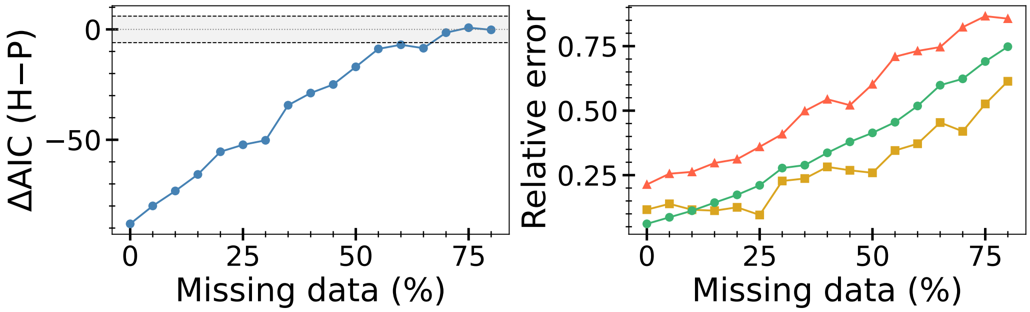

Figure 7: **Method remains reliable up to approximately 50% missing data for bursty processes.** A single Hawkes series of $N_{\text{e}} = 300$ events with true parameters $(\lambda_0^*, \alpha^*, \delta^*) = (1, 3, 7)$ is thinned by randomly removing a fraction q of events; both models are then refit to the thinned series. *Left:* $\Delta\text{AIC} = \text{AIC(Hawkes)} - \text{AIC(Poisson)}$ as a function of missing data percentage. Black dashed lines: ± 6 (95% confidence thresholds). Gray band: inconclusive region. Dotted horizontal line: $\Delta\text{AIC} = 0$. *Right:* Relative parameter estimation error $|\hat{\theta} - \theta^*|/\theta^*$ for λ_0 (green circles), α (red triangles), and δ (gold squares) (ten trial averages).

We note that the robustness of the method depends on how clustered (bursty) the underlying process truly is. For the more bursty parameter set shown in Fig 7, the method tolerates up to approximately 60% missing data before model selection becomes unreliable. For a less bursty process — for example $(\lambda_0^*, \alpha^*, \delta^*) = (1, 2, 3.5)$, which has a lower branching ratio α/δ — the corresponding crossover occurs earlier, at approximately $q = 40\%$ for model selection and $q = 30\%$ for reliable parameter estimation (Relative errors $< 50\%$). In both cases, the method remains robust to moderate reporting gaps, and the threshold is well above the missing data rate expected in the SCD cohort, where data collection was actively supported through app notifications and regular staff follow-ups.

These results establish that the method is robust to moderate reporting gaps, with the precise tolerance depending on the burstiness of the underlying process[2]. In the SCD cohort, the data

[2]In general, more bursty processes retain stronger self-excitation signal even after thinning, making them more robust to missing data.

were collected with active clinical encouragement to report daily, so the effective missing data rate is expected to be considerably lower than the degradation threshold in either parameter regime. Moreover, since a pain level of zero is defined as a non-event, the event timestamps directly correspond to reported pain, preserving the underlying signal dynamics rather than reflecting reporting artifacts.

8 Summary

Taken together, the experiments in this appendix establish two conclusions. First, our KS based augmentation method performs its intended function: it improves model selection when series share a common process and does not do so when they do not, and parameter estimates remain stable under single-member removal from the augmented group and under moderate levels of missing data. *These results indicate that, when the similarity threshold p_c selects genuinely similar series, the overall augmentation procedure yields reliable and robust inference.*

Second, automatic selection of p_c is non-trivial: the optimal threshold depends on series length, mean rate separation, and distributional geometry in ways that interact, and the criterion may be blind to differences in self-excitation structure at very small data sizes ($n \approx 30$). *In particular, the mapping between distributional similarity and KS p-values is itself sample-size dependent, and may not admit a uniquely optimal threshold in finite data regimes.* Addressing this rigorously—connecting it to power analysis, information-theoretic criteria, or community detection on the p-value graph—is an important open problem for future research.
