# Supplementary material for: A new method for augmenting short time series, with application to pain events in sickle cell disease
Source: PLoS Comput Biol. 2026 Jun 12;22(6):e1014389. doi: 10.1371/journal.pcbi.1014389 (PMC13286270; doi:10.1371/journal.pcbi.1014389)
Supplement: Attachment — Submitted filename: Response to PLOS CB.docx [file pcbi.1014389.s007.docx]

Manuscript ID: PCOMPBIOL-D-26-00088

Manuscript Title: A new method for augmenting short time series, with application to pain events in sickle cell disease

Journal: PLOS Computational Biology

Comments from academic editor:

Dear Dr. Utkarsh,

Thank you for submitting your manuscript to PLOS Computational Biology. After careful consideration, we feel that it has merit but does not fully meet PLOS Computational Biology's publication criteria as it currently stands. Therefore, we invite you to submit a revised version of the manuscript that addresses the points raised during the review process.

…

Kind regards,

Lun Hu

Academic Editor

PLOS Computational Biology

Denise Kühnert

Section Editor

PLOS Computational Biology

–-

Additional Editor Comments:

I received three review reports, and all reviewers found merit in this manuscript. However, they also raised several critical concerns, such as the clarification of motivation, the extension of validation on robustness and statistical interpretation, the discussion of key limitations, the scalability, and the memory usage. Due to these issues, I would like to ask authors to revise their manuscript for major revision.

Dear Dr. Hu and Dr. Kühnert,

We were happy to read that the reviewers all found merit in our manuscript and thank you for the opportunity to respond to their concerns. We have now made significant revisions to our manuscript (both clarifications and additional explanations and extensions), and we believe it is much improved. Please find our detailed responses to reviewer comments below.

We thank you once again for your consideration.

Sincerely,

Kumar Utkarsh and Daniel M. Abrams

*(On behalf of all co-authors)*

.

**Responses to Reviewer #1:**

*This article presents a strategy for what they call “data augmentation” with the intent of creating better models with sparse data sets with time series data. It is a very important and relevant problem, one I encounter in my own work frequently. I thought the article was well written and should be published pending some revisions. My general observations are that the work addresses an important topic, but I think it might be re-inventing the wheel a little, since I believe a similar the problem is well-studied in other fields under different nomenclature (as far I as I can understand). My specific technical comments follow. Otherwise, I see other no red flags in the way the work was conducted technically speaking. The authors have provided much supplemental theory and data as well. I think it was a very nice work.*

We thank Reviewer #1 for the detailed feedback and kind comments and are glad that they found the topic important and relevant. We are also happy that they recommend publication (pending revision).

*1. I am not sure “data augmentation” is the right term for what you are doing. For example, in the intro, augmentation is described as “generation of synthetic data points”, which in my domain are referred to as “space filling algorithms”. Those space filling algorithms are used in my field frequently for the explicit purpose of “dynamical systems with … inherent strong temporal correlation” as you say in the intro as having received little attention. I think you should do a quick literature check on space filling algorithms for dynamic models, dynamic optimization, or dynamic model fitting and make sure that you are not re-inventing the wheel.*

We agree with the reviewer’s comment in the sense that the term "augmentation" is not used by us in the sense of synthesizing new data — we did not mean the generation of synthetic data points, but rather the pooling of multiple existing datasets through a collective likelihood (i.e., leveraging the simultaneous use of multiple existing datasets). We have revised the introduction accordingly to make the distinction clearer. Regarding space-filling algorithms: we have reviewed this literature and note that such methods are primarily concerned with optimal experimental design, selecting new sampling locations to cover a parameter or state space efficiently. Our problem is different from that: we have no control over sampling and instead seek to improve inference from fixed, sparse observations. We now cite [McKay et al 1979, Wickle et al 2019] as representative of this literature.

*2. Your approach with Hawkes models is very similar to that of Kriging models, which are well known in my domain. It seems Kriging models are to the space domain as Hawkes models are to the time domain. Both can use exponential basis functions as you have done here. However, there are many studies which extend Kriging to the time domain for dynamic models. It seems really close to what you have here. So, I think you need to check that out as well.*

We appreciate the reviewer pointing us to this literature. While both frameworks (Kriging and Hawkes) can employ exponential kernel functions, the objectives differ fundamentally: Kriging-based methods are designed for spatial or spatio-temporal interpolation — predicting field values at unobserved locations — whereas our method performs likelihood-based inference for event processes without interpolation. In our specific application to sickle cell disease, we assume zero pain between observed reports rather than interpolating pain levels, which is our chosen clinical assumption for self-report data. We have added a note in paragraph 11 of the revised manuscript’s Discussion section to acknowledge this connection, along with corresponding citations.

*3. The only technical problem with the implementation is a seeming lack of model validation (unless I have really missed something). Normally, for model development generally, we need to test the model on a data set different from what it was trained. There is no doubt to your result that Hawkes fits better than Poisson. However, there is the possibility that the Hawkes model is better by only simple virtue of having more parameters available to fit (e.g. overfitting or spurious fitting). With testing, you can show that the Hawkes model has predictive power as well because it captures at least some amount of the information found from different samples at different time points that are common to all the samples (e.g. the same underlying model/physical principle) that Poisson does not. Given your sparse data problem, this is not a trivial thing to do. An alternative postulation then is a more meta approach. Show that given completely unrelated data (e.g. comparing time-series apples to time-series oranges), the Hawkes model does not improve over Poisson at all, or if it does, it improves by a small amount. (E..g the Hawkes improvement of your actual SCD patients is far greater over Poisson than when using unrelated data). Then you can show at least at a meta-level the degree at which having more parameters is attributed to model fitting on its own right.*

We thank the reviewer for this important methodological point. We address it in two parts.

First, AIC explicitly penalizes model complexity — our Hawkes model carries a penalty for three additional parameters relative to Poisson. A preference for Hawkes under AIC therefore cannot be attributed solely to parameter count.

Second, we note that formal cross-validation is not directly applicable in our setting. Our inference framework is likelihood-based: the likelihood measures goodness-of-fit of a model to observed data and is used for model comparison, not prediction on held-out sets. There is no natural separation into training and test data, and with sparse event series, further partitioning would make the estimation problem even less reliable. In a data-abundant setting, one could hold out a continuous segment, estimate parameters on the remainder, and compare out-of-sample log-likelihoods between Hawkes and Poisson on the held-out segment; the dependence of the Hawkes likelihood on event history across the split boundary, combined with data sparsity, makes this unreliable here.

Instead, we attempt to address the spirit of the reviewer's concern (could augmentation artificially inflate preference for the more complex model?) through a new negative control experiment described in S4 Appendix Sec. 6 (see Fig. 6 in particular). We apply the augmentation pipeline to two conditions: (A) series drawn from the same Hawkes process (matched, positive control), and (B) series drawn from Hawkes processes with deliberately dissimilar mean rates, spanning over an order of magnitude (unrelated, negative control). If augmentation were inflating Hawkes preference as an artifact of pooling, we would expect similar ΔAIC shifts in both conditions. Instead, we find that the ΔAIC improvement under augmentation is large and consistent for the matched condition, while remaining negligible for the unrelated condition. This demonstrates (at least in the case of this example) that the method responds to genuine shared structure rather than to the act of pooling itself, and that the improvement observed in SCD patients cannot be attributed to a parameter-count artifact.

*4. You have the key limitation of “Key limitation. For the augmentation method to be applicable, there must be a reasonable expectation that multiple datasets came from the same (or very similar) models.”. Actually I don’t think that is a limitation, but rather the underlying theoretical principle under which this approach works. So I don’t think you should diminish this by calling it a limitation. I frankly don’t know how it would be meaningful to connect any unrelated data in this manner regardless of algorithm otherwise. You speak of the underlying “models parameters” and “governing equations” but frankly I think that is not as meaningful as talking about the requirement that the samples are governed by the same underlying physical phenomena.*

We agree with the reviewer's reframing and have revised the Discussion accordingly. What we previously labeled a "key limitation" is more accurately described as a foundational operating condition for the method: meaningful augmentation requires that the pooled datasets share a common generative process. We have reworded this passage (Discussion, paragraph 3) to reflect this, and have replaced "limitation" with language emphasizing that this is the theoretical basis for the approach rather than a constraint on its validity.

**Responses to Reviewer #2:**

*The manuscript presents a similarity-based data augmentation strategy for sparse event time series, motivated by patient-reported pain events in sickle cell disease. The paper is generally readable and the narrative is coherent, especially in connecting sparse data limitations to Hawkes-vs-Poisson discrimination. Several aspects of methodological rigor, statistical validity, and reproducibility require strengthening before the work is ready for publication.*

We thank Reviewer #2 for the detailed feedback. We have made significant modifications to the manuscript in response to their comments and hope that they now see it as ready for publication.

*1.The framing of the introduction should be sharpened. Please state explicitly whether the manuscript targets robust model selection/parameter estimation for extremely sparse point processes or a more general cross-subject information-sharing paradigm, and articulate the core contribution in a concise, falsifiable form.*

We thank the reviewer for this suggestion. The manuscript targets both goals simultaneously — robust model selection and parameter estimation for sparse point processes, achieved through a principled cross-subject information-sharing framework. We have revised the final paragraph of the introduction to state this explicitly and added a concise summary of the core contribution.

*2.The motivation for the similarity definition needs to be made explicit. The method operationalizes “series similarity” via a KS test on interarrival-time distributions, yet dependence in Hawkes-type processes can affect interarrival behavior; the manuscript should justify why this proxy is appropriate and delineate its anticipated failure modes.*

Our choice to assess series similarity via the two-sample KS test (applied to interarrival-time distributions) is motivated directly by the structure of our model selection problem: the Poisson null model is characterized by independent exponential interarrivals, while the Hawkes alternative introduces dependence between successive gaps through self-excitation. Interarrival distributions therefore capture precisely the statistical property that distinguishes the two candidate models, making them the natural model-free feature for assessing whether two series share the same underlying process.

The KS test is appealing because it is nonparametric — it makes no assumption about the functional form of the interarrival distribution, which has no simple closed form for Hawkes processes. Yet it is sensitive to differences in both distribution location and shape, and is computationally trivial to apply pairwise across a collection.

In our revised manuscript, we now compare the KS test to the Anderson-Darling test, which has greater sensitivity to tail differences, and we find comparable precision, recall, and F1 at our typical sample sizes (please see the first section of the new S4 Appendix), suggesting our conclusions are not sensitive to this methodological choice. The robustness is further supported empirically by the addition of a negative control experiment we described in S4 Appendix Section 7: augmentation of series drawn from unrelated Hawkes parameter regimes did not improve model selection, indicating that the KS test successfully avoids grouping series with meaningfully different dependence structures.

In addition to the new supplementary document (S4), we have now added further clarification and discussion of our use of the KS test in the second paragraph of the revised “Data Augmentation” subsection in the manuscript’s “Materials and methods” section.

*3.The choice of the KS significance threshold pcp_cpc lacks a principled specification. The current description appears heuristic; please provide a clear, reproducible rule for selecting pcp_cpc (and clarify whether it depends on sample size or desired graph sparsity), and explain how this choice influences the resulting match graph.*

We agree that the choice of p_c lacks a principled specification and that this is a true limitation arising out of the complexity of the problem. The optimal threshold depends, among many other possible factors, on series length, mean rate separation, and distributional geometry in ways that interact, making a single universal rule unavailable. In our revised manuscript, we have added several sections to the new S4 Appendix where we attempt to characterise this dependence systematically. In brief, the threshold controls a tradeoff between true positive and true negative rates that shifts with sample size (S4 Appendix Sec. 3-4), and automatic threshold selection connects to power analysis and community detection on the p-value graph — a problem we identify as a meaningful open question reserved for future work (S4 Appendix Sec. 8). For the present manuscript we retain p_c=0.10 as a reasonable heuristic, supported by robustness experiments presented in S4 Appendix Sec. 6-7.

*4.The statistical implications of non-transitive matching are under-specified. Given that matches need not be transitive, the manuscript should clarify how the “neighborhood” used for augmentation is defined (e.g., one-hop neighbors vs. connected components) and how this choice affects the strength and potential bias of information sharing.*

We thank the reviewer for this clarifying question. The neighbourhood used for augmentation of series $j$ is defined explicitly as the set of one-hop neighbours in the KS similarity graph:

$N(j)=\{i\neq j:p(i,j)\geq p_{c}\}$, where $p(i,j)$ is the p-value for the interarrival distributions of series $i$ and $j$. No transitive closure or connected-component construction is applied. Each series receives its own independent neighbourhood and its own independent collective likelihood and parameter fit. We now explicitly state this point in Step 3 of our augmentation strategy definition (in the Materials and Methods section of the manuscript).

Non-transitivity is a feature rather than an ambiguity: it allows each series to be augmented only by those series that are directly and empirically similar to it, without inheriting similarity relationships it does not directly satisfy. This is illustrated in Fig. 3 in our main text, where series 1 is similar to both 2 and 3, but 2 and 3 are dissimilar to each other. Under our approach, augmenting series 2 includes series 1 but not series 3, and similarly, augmenting series 3 includes series 1 but not series 2. The alternative — connected-component pooling — would force 2 and 3 into the same group via their shared neighbour 1, potentially introducing bias by pooling “dissimilar” series.

*5.The collective (product-form) likelihood requires a clearer statistical interpretation. Since the proposed likelihood effectively treats matched peers as additional weighted evidence, please make the implicit assumptions (e.g., approximate exchangeability or near-identical generative mechanisms within a neighborhood) explicit and distinguish this construction conceptually from hierarchical/partial-pooling formulations.*

The collective likelihood makes one key assumption: all series in an augmentation group are independent realizations of the same underlying process with identical parameters. Conceptually, this is equivalent to observing one longer realization of the same process.

In our formulas, we are simply extending the product of probability densities over interarrival times from one series to multiple series, with the same parameter vector appearing in every factor of the likelihood expression. This is strictly stronger than hierarchical or partial-pooling formulations, which allow parameters to vary across group members and share only a hyperprior. Our construction makes no use of hyperpriors.

This assumption is straight-forward, testable (via the KS grouping step), and deliberately strong — if it is violated the method will produce biased estimates, which is why the similarity screening step is essential. We have now made the above arguments explicit in the last paragraph of the “Data Augmentation” subsection of the “Materials and methods” section in our revised manuscript.

*6.The authors propose an effective strategy. In future work, widely developed deep learning techniques and their application to more complex scenarios (DOI: 10.1145/3664647.3681673, DOI: 10.1109/JBHI.2024.3357979) could be further explored.*

We thank the reviewer for pointing us to these techniques that can also be applicable to problems like the one we explore in our manuscript. We have now cited these papers and mentioned the potential complementary applicability in the “Future Directions” part of our revised Discussion section.

**Responses to Reviewer #3:**

*This manuscript presents a novel data augmentation strategy for analyzing sparse temporal event data by pooling statistically similar short time series and fitting models using a collective likelihood framework. The method is systematically validated on synthetic data for distinguishing Hawkes and Poisson processes and is then applied to patient-reported pain events in sickle cell disease, demonstrating improved model selection and parameter estimation compared to single-series analysis. The approach is technically sound and addresses an important practical limitation in real-world biomedical time series analysis, with potential applicability beyond the specific case study. However, the validity of key assumptions underlying similarity-based pooling and the robustness of the method to mis-grouping and model misspecification require clearer justification and additional validation.*

We thank Reviewer #3 for the detailed feedback and see their summary as appropriate and accurate. We have attempted to strengthen our presentation of key assumptions and assessment of the robustness of our method in the revised manuscript, and we hope this addresses their concerns.

*Questions:
1. How robust is the proposed augmentation method when time series generated from different underlying Hawkes parameter regimes are incorrectly grouped as “similar”?*

This is an important point that we did not sufficiently address in our initial submission, as evidenced by all three reviewers highlighting it! We believe that our revised manuscript is now much more direct about the applicability and limitations of our augmentation method, and we have also added a significant amount of new material addressing this point in our new S4 Appendix. In particular, S4 Appendix Section 6 discusses a negative control experiment in which we examine exactly the issue of robustness to differing Hawkes parameter regimes. The ΔAIC improvement from augmentation is large for matched series, but substantially reduced for unrelated series whose parameters were chosen to be different deliberately. This confirms that the improvement requires genuine shared structure among pooled series and is not an artifact of pooling per se.

In other sections of that appendix, we also test the robustness of our method to alternative metrics for dataset similarity, to the choice of threshold *p_c_*, and to the omission of significant amounts of data.

*2. What is the false positive rate for detecting Hawkes dynamics when all underlying processes are truly Poisson but augmented using the proposed framework?*

The brief answer is about 10%. We now discuss this risk and present results of a numerical experiment in the latter part of the revised manuscript’s S4 Appendix Section 6 (see Table 1 and preceding text). We ran 100 collections of 10 pure Poisson series and applied the full augmentation pipeline. The single-series FPR is negligible (0.001 at the tight threshold). The augmented FPR rises to 0.098, because pooling increases statistical power indiscriminately when the KS test cannot distinguish Poisson from Hawkes interarrival distributions at n=30. We therefore recommend the method be used in settings where self-exciting dynamics are plausible a priori rather than as a conservative test of the Poisson null.

*3. Why was the two-sample KS test on interarrival time distributions chosen as the similarity metric, and how does it compare with alternative distance measures on event sequences?*

This question was also posed by Reviewer #2. The KS test was chosen because it is nonparametric, sensitive to both location and shape differences, and requires no distributional assumptions. In the revised manuscript’s new S4 Appendix, Section 1, we now compare performance directly between the KS test and the Anderson-Darling two-sample test. Both perform similarly, though KS is slightly better on precision, recall, and F1 at the sample sizes present in our data. We expect that our results would apply similarly to other comparable distance measures as well.

*4. How sensitive are the inferred model selection outcomes and parameter estimates to the choice of the similarity threshold pc?*

This question connects to some of the previous ones posed by both Reviewer #2 and Reviewer #3. To address it we have now performed explicit sensitivity analyses as described in the revised manuscript’s S4 Appendix Sections 3, 4, and 5. The series length dependencies (S4 Sec. 3) show that *p_c_* affects model selection in an intermediate regime between clearly identical and clearly disjoint series — the regime our data occupies. S4 Sec. 4 further shows that the KS criterion is very weakly sensitive to branching ratio differences at *N_e_* = 30, though it should be able to distinguish differences in mean event rates. Parameter estimate sensitivity is addressed in S4 Sec. 5 (see also our response to Question 6 below).

*5. Can the authors provide a principled strategy or guideline for choosing pc in practice?*

Developing a generally applicable and theoretically grounded method for choosing *p_c_* is an important problem, however, it is one that we believe is beyond the scope of the current manuscript. S4 Appendix Sections 3-4 characterise how the threshold's discriminative power depends on series length and distributional distance, establishing the boundary conditions within which *p_c_* = 0.10 is a reasonable heuristic. We would like to also develop an automatic method for optimizing this threshold, but expect it to require careful analyses connecting statistical power, information theory, and community detection algorithms (on the p-value matrix). This is ongoing research that we would prefer to leave for future work, as we now state in the revised manuscript’s S4 Appendix Sec. 8.

*6. How stable are the Hawkes parameter estimates (α, δ) under small changes in the composition of the augmented groups?*

In the revised manuscript’s S4 Appendix Section 5, we now assess the stability of parameter estimates under changes in group composition via a leave-one-out analysis. Coefficients of variation for α, δ, and the branching ratio α/δ all remain below 0.10 across focal series, confirming that estimates are fairly robust with respect to small changes in the composition of the augmented group. The decay rate δ shows the greatest sensitivity, consistent with the known flatness of the likelihood surface in the α–δ direction (see S2 Appendix). The branching ratio α/δ, which governs self-excitation strength and is the quantity of primary clinical interest, is the most stable.

*7. The inferred Hawkes memory timescales for SCD pain events appear to be on the order of minutes. How should these timescales be interpreted physiologically, given that pain crises typically evolve over much longer periods?*

We have expanded the Discussion section of our revised manuscript to address this (see paragraph 13). Pain crises and individual pain events as captured by the SMART app are distinct — a crisis may span days but individual reported events reflect momentary pain intensity above baseline. Shorter timescales changes may be driven by physiological processes such as inflammatory dynamics and blood pressure regulation that operate on minute-to-hour timescales; the short memory timescales we infer likely reflect these faster fluctuations. We also note that with only weeks to months of data per patient we may lack power to detect longer-timescale memory effects even if present, and that denser longer-term datasets may reveal memory at multiple timescales.

*8. Could the short memory timescales be an artifact of the event definition and reporting frequency rather than true underlying dynamics?*

This is possible in principle, but we believe it is unlikely to be a significant concern for this dataset for two reasons. First, data collection was actively supported — app notifications prompted daily reporting and nursing staff encouraged reporting both through remote interaction (text messages, phone calls) and during hospital visits — so the reported events are expected to closely reflect true pain occurrences. Second, a pain level of zero constitutes a non-event by our definition, meaning the event timestamps are precisely the pain reports themselves. The dynamics of the reported signal are therefore the same as the dynamics of the pain, not an artifact of the reporting protocol. To the extent that some events were nonetheless missed, our sensitivity analysis (S4 Appendix Sec. 7) shows the method remains reliable up to approximately 30–40% missing data, well above the missing rate expected in this cohort.

*9. How does reporting fatigue or missing entries in the SMART app data affect the inferred self-exciting dynamics, and has this been tested via sensitivity analyses?*

This is an interesting question that we thank Reviewer #3 for asking. In our revised manuscript, we now report on a numerical experiment we have performed to answer it. As discussed in S4 Appendix Section 7, we randomly remove *q*% of events from a synthetic Hawkes series (*N_e_* = 300, (λ,α,δ)=(1,3,7)) and refit both Hawkes and Poisson models. Hawkes is confidently preferred up to approximately *q* = 60% missing data, and parameter errors remain below 0.5 for about *q* <≈ 50%. The robustness of the method depends on how clustered the underlying process truly is, the more clustered a process is the more robust it is to missing data. In the SCD cohort, data were collected with active clinical encouragement to report daily, so the effective missing rate is expected to be well below the degradation threshold.

*10. Given known limitations of AIC for dynamical systems, did the authors evaluate whether conclusions are robust to alternative model selection criteria.*

We have considered this carefully and in fact have written a separate manuscript (which is currently under review itself) examining conditions under which AIC fails for dynamical systems (<https://doi.org/10.48550/arXiv.2511.14931>). However we do not believe this is a material concern here — the standard regularity conditions underlying AIC are well satisfied for the Hawkes and Poisson point process likelihoods, which are smooth and identifiable in the model selection sense. Our SI already includes an AICc comparison (S3 Appendix) reaching identical conclusions.

**Competing interests:** The authors have declared that no competing interests exist.

**Potential Copyright Issues (Figure 6):** Since the application is self-developed, we do not need to seek external permission for the application logo. We provide the appropriate citation in the caption of the figure. We have removed the other branding name from the figure.
