# Supplementary material for: A new method for augmenting short time series, with application to pain events in sickle cell disease
Source: PLoS Comput Biol. 2026 Jun 12;22(6):e1014389. doi: 10.1371/journal.pcbi.1014389 (PMC13286270; doi:10.1371/journal.pcbi.1014389)
Supplement: Peer review history

# Peer review: Decision Letter 0

**DOI:** 10.1371/journal.pcbi.1014389.r001

**Authors:** Lun Hu, Denise Kühnert

11 Mar 2026

PCOMPBIOL-D-26-00088

A new method for augmenting short time series, with application to pain events in sickle cell disease

PLOS Computational Biology

Dear Dr. Utkarsh,

Thank you for submitting your manuscript to PLOS Computational Biology. After careful consideration, we feel that it has merit but does not fully meet PLOS Computational Biology's publication criteria as it currently stands. Therefore, we invite you to submit a revised version of the manuscript that addresses the points raised during the review process.

Please submit your revised manuscript by May 11 2026 11:59PM. If you will need more time than this to complete your revisions, please reply to this message or contact the journal office at ploscompbiol@plos.org. When you're ready to submit your revision, log on to https://www.editorialmanager.com/pcompbiol/ and select the 'Submissions Needing Revision' folder to locate your manuscript file.

Please include the following items when submitting your revised manuscript:

* A letter that responds to each point raised by the editor and reviewer(s). You should upload this letter as a separate file labeled 'Response to Reviewers'. This file does not need to include responses to formatting updates and technical items listed in the 'Journal Requirements' section below.

* A marked-up copy of your manuscript that highlights changes made to the original version. You should upload this as a separate file labeled 'Revised Manuscript with Track Changes'.

* An unmarked version of your revised paper without tracked changes. You should upload this as a separate file labeled 'Manuscript'.

If you would like to make changes to your financial disclosure, competing interests statement, or data availability statement, please make these updates within the submission form at the time of resubmission. Guidelines for resubmitting your figure files are available below the reviewer comments at the end of this letter

We look forward to receiving your revised manuscript.

Kind regards,

Lun Hu

Academic Editor

PLOS Computational Biology

Denise Kühnert

Section Editor

PLOS Computational Biology

**Additional Editor Comments:**

I received three review reports, and all reviewers found merit in this manuscript. However, they also raised several critical concerns, such as the clarification of motivation, the extension of validation on robustness and statistical interpretation, the discussion of key limitations, the scalability, and the memory usage. Due to these issues, I would like ask authors to revise their manuscript for major revision.

**Journal Requirements:**

If the reviewer comments include a recommendation to cite specific previously published works, please review and evaluate these publications to determine whether they are relevant and should be cited. There is no requirement to cite these works unless the editor has indicated otherwise.

1) We ask that a manuscript source file is provided at Revision. Please upload your manuscript file as a .doc, .docx, .rtf or .tex. If you are providing a .tex file, please upload it under the item type u2018LaTeX Source Fileu2019 and leave your .pdf version as the item type u2018Manuscriptu2019.

2) Please upload all main figures as separate Figure files in .tif or .eps format. For more information about how to convert and format your figure files please see our guidelines:

https://journals.plos.org/ploscompbiol/s/figures

3) Some material included in your submission may be copyrighted. According to PLOSu2019s copyright policy, authors who use figures or other material (e.g., graphics, clipart, maps) from another author or copyright holder must demonstrate or obtain permission to publish this material under the Creative Commons Attribution 4.0 International (CC BY 4.0) License used by PLOS journals. Please closely review the details of PLOSu2019s copyright requirements here: PLOS Licenses and Copyright. If you need to request permissions from a copyright holder, you may use PLOS's Copyright Content Permission form.

Please respond directly to this email and provide any known details concerning your material's license terms and permissions required for reuse, even if you have not yet obtained copyright permissions or are unsure of your material's copyright compatibility. Once you have responded and addressed all other outstanding technical requirements, you may resubmit your manuscript within Editorial Manager.

Potential Copyright Issues:

i) Figure 6. Please confirm whether you drew the images / clip-art within the figure panels by hand. If you did not draw the images, please provide (a) a link to the source of the images or icons and their license / terms of use; or (b) written permission from the copyright holder to publish the images or icons under our CC BY 4.0 license. Alternatively, you may replace the images with open source alternatives. See these open source resources you may use to replace images / clip-art:

- https://commons.wikimedia.org

- https://openclipart.org/.

ii) The following Figure contains a logo or branding: 6. We are not permitted to publish this under our CC-BY 4.0 license, even with permission. We ask that you please remove or replace it.

4) Please send a completed 'Competing Interests' statement, including any COIs declared by your co-authors. If you have no competing interests to declare, please state "The authors have declared that no competing interests exist". Otherwise please declare all competing interests beginning with the statement "I have read the journal's policy and the authors of this manuscript have the following competing interests"

**Reviewers' comments:**

Reviewer's Responses to Questions

**Comments to the Authors:**

**Please note here if the review is uploaded as an attachment.**

Reviewer #1: This article presents a strategy for what they call “data augmentation” with the intent of creating better models with sparse data sets with time series data. It is a very important and relevant problem, one I encounter in my own work frequently. I thought the article was well written and should be published pending some revisions. My general observations are that the work addresses an important topic, but I think it might be re-inventing the wheel a little, since I believe a similar the problem is well-studied in other fields under different nomenclature (as far I as I can understand). My specific technical comments follow. Otherwise, I see other no red flags in the way the work was conducted technically speaking. The authors have provided much supplemental theory and data as well. I think it was a very nice work.

1. I am not sure “data augmentation” is the right term for what you are doing. For example, in the intro, augmentation is described as “generation of synthetic data points”, which in my domain are referred to as “space filling algorithms”. Those space filling algorithms are used in my field frequently for the explicit purpose of “dynamical systems with … inherent strong temporal correlation” as you say in the intro as having received little attention. I think you should do a quick literature check on space filling algorithms for dynamic models, dynamic optimization, or dynamic model fitting and make sure that you are not re-inventing the wheel.

2. Your approach with Hawkes models is very similar to that of Kriging models, which are well known in my domain. It seems Kriging models are to the space domain as Hawkes models are to the time domain. Both can use exponential basis functions as you have done here. However, there are many studies which extend Kriging to the time domain for dynamic models. It seems really close to what you have here. So, I think you need to check that out as well.

3. The only technical problem with the implementation is a seeming lack of model validation (unless I have really missed something). Normally, for model development generally, we need to test the model on a data set different from what it was trained. There is no doubt to your result that Hawkes fits better than Poisson. However, there is the possibility that the Hawkes model is better by only simple virtue of having more parameters available to fit (e.g. overfitting or spurious fitting). With testing, you can show that the Hawkes model has predictive power as well because it captures at least some amount of the information found from different samples at different time points that are common to all the samples (e.g. the same underlying model/physical principle) that Poisson does not. Given your sparse data problem, this is not a trivial thing to do. An alternative postulation then is a more meta approach. Show that given completely unrelated data (e.g. comparing time-series apples to time-series oranges), the Hawkes model does not improve over Poisson at all, or if it does, it improves by a small amount. (E..g the Hawkes improvement of your actual SCD patients is far greater over Poisson than when using unrelated data). Then you can show at least at a meta-level the degree at which having more parameters is attributed to model fitting on its own right.

4. You have the key limitation of “Key limitation. For the augmentation method to be applicable, there must be a reasonable expectation that multiple datasets came from the same (or very similar) models.”. Actually I don’t think that is a limitation, but rather the underlying theoretical principle under which this approach works. So I don’t think you should diminish this by calling it a limitation. I frankly don’t know how it would be meaningful to connect any unrelated data in this manner regardless of algorithm otherwise. You speak of the underlying “models parameters” and “governing equations” but frankly I think that is not as meaningful as talking about the requirement that the samples are governed by the same underlying physical phenomena.

Reviewer #2: The manuscript presents a similarity-based data augmentation strategy for sparse event time series, motivated by patient-reported pain events in sickle cell disease. The paper is generally readable and the narrative is coherent, especially in connecting sparse data limitations to Hawkes-vs-Poisson discrimination. Several aspects of methodological rigor, statistical validity, and reproducibility require strengthening before the work is ready for publication.

1.The framing of the introduction should be sharpened. Please state explicitly whether the manuscript targets robust model selection/parameter estimation for extremely sparse point processes or a more general cross-subject information-sharing paradigm, and articulate the core contribution in a concise, falsifiable form.

2.The motivation for the similarity definition needs to be made explicit. The method operationalizes “series similarity” via a KS test on interarrival-time distributions, yet dependence in Hawkes-type processes can affect interarrival behavior; the manuscript should justify why this proxy is appropriate and delineate its anticipated failure modes.

3.The choice of the KS significance threshold pcp_cpc lacks a principled specification. The current description appears heuristic; please provide a clear, reproducible rule for selecting pcp_cpc (and clarify whether it depends on sample size or desired graph sparsity), and explain how this choice influences the resulting match graph.

4.The statistical implications of non-transitive matching are under-specified. Given that matches need not be transitive, the manuscript should clarify how the “neighborhood” used for augmentation is defined (e.g., one-hop neighbors vs. connected components) and how this choice affects the strength and potential bias of information sharing.

5.The collective (product-form) likelihood requires a clearer statistical interpretation. Since the proposed likelihood effectively treats matched peers as additional weighted evidence, please make the implicit assumptions (e.g., approximate exchangeability or near-identical generative mechanisms within a neighborhood) explicit and distinguish this construction conceptually from hierarchical/partial-pooling formulations.

6.The authors propose an effective strategy. In future work, widely developed deep learning techniques and their application to more complex scenarios (DOI: 10.1145/3664647.3681673, DOI: 10.1109/JBHI.2024.3357979) could be further explored.

Reviewer #3: Summary

This manuscript presents a novel data augmentation strategy for analyzing sparse temporal event data by pooling statistically similar short time series and fitting models using a collective likelihood framework. The method is systematically validated on synthetic data for distinguishing Hawkes and Poisson processes and is then applied to patient-reported pain events in sickle cell disease, demonstrating improved model selection and parameter estimation compared to single-series analysis. The approach is technically sound and addresses an important practical limitation in real-world biomedical time series analysis, with potential applicability beyond the specific case study. However, the validity of key assumptions underlying similarity-based pooling and the robustness of the method to mis-grouping and model misspecification require clearer justification and additional validation.

Questions:

1. How robust is the proposed augmentation method when time series generated from different underlying Hawkes parameter regimes are incorrectly grouped as “similar”?

2. What is the false positive rate for detecting Hawkes dynamics when all underlying processes are truly Poisson but augmented using the proposed framework?

3. Why was the two-sample KS test on interarrival time distributions chosen as the similarity metric, and how does it compare with alternative distance measures on event sequences?

4. How sensitive are the inferred model selection outcomes and parameter estimates to the choice of the similarity threshold pc?

5. Can the authors provide a principled strategy or guideline for choosing pc in practice?

6. How stable are the Hawkes parameter estimates (α, δ) under small changes in the composition of the augmented groups?

7. The inferred Hawkes memory timescales for SCD pain events appear to be on the order of minutes. How should these timescales be interpreted physiologically, given that pain crises typically evolve over much longer periods?

8. Could the short memory timescales be an artifact of the event definition and reporting frequency rather than true underlying dynamics?

9. How does reporting fatigue or missing entries in the SMART app data affect the inferred self-exciting dynamics, and has this been tested via sensitivity analyses?

10. Given known limitations of AIC for dynamical systems, did the authors evaluate whether conclusions are robust to alternative model selection criteria.

**********

**Have the authors made all data and (if applicable) computational code underlying the findings in their manuscript fully available?**

The PLOS Data policy requires authors to make all data and code underlying the findings described in their manuscript fully available without restriction, with rare exception (please refer to the Data Availability Statement in the manuscript PDF file). The data and code should be provided as part of the manuscript or its supporting information, or deposited to a public repository. For example, in addition to summary statistics, the data points behind means, medians and variance measures should be available. If there are restrictions on publicly sharing data or code —e.g. participant privacy or use of data from a third party—those must be specified.

Reviewer #1: None

Reviewer #2: Yes

Reviewer #3: Yes

**********

PLOS authors have the option to publish the peer review history of their article (what does this mean?). If published, this will include your full peer review and any attached files.

If you choose “no”, your identity will remain anonymous but your review may still be made public.

**Do you want your identity to be public for this peer review?**   For information about this choice, including consent withdrawal, please see our Privacy Policy.

Reviewer #1: No

Reviewer #2: No

Reviewer #3: No

**Figure resubmission:**

While revising your submission, we strongly recommend that you use PLOS’s NAAS tool (https://ngplosjournals.pagemajik.ai/artanalysis) to test your figure files. NAAS can convert your figure files to the TIFF file type and meet basic requirements (such as print size, resolution), or provide you with a report on issues that do not meet our requirements and that NAAS cannot fix.

After uploading your figures to PLOS’s NAAS tool - https://ngplosjournals.pagemajik.ai/artanalysis, NAAS will process the files provided and display the results in the "Uploaded Files" section of the page as the processing is complete. If the uploaded figures meet our requirements (or NAAS is able to fix the files to meet our requirements), the figure will be marked as "fixed" above. If NAAS is unable to fix the files, a red "failed" label will appear above. When NAAS has confirmed that the figure files meet our requirements, please download the file via the download option, and include these NAAS processed figure files when submitting your revised manuscript.

**Reproducibility:**

To enhance the reproducibility of your results, we recommend that authors of applicable studies deposit laboratory protocols in protocols.io, where a protocol can be assigned its own identifier (DOI) such that it can be cited independently in the future. Additionally, PLOS ONE offers an option to publish peer-reviewed clinical study protocols. Read more information on sharing protocols at https://plos.org/protocols?utm_medium=editorial-email&utm_source=authorletters&utm_campaign=protocols

# Author comment: Author response to Decision Letter 1

**DOI:** 10.1371/journal.pcbi.1014389.r002

14 May 2026

Attachment Submitted filename: Response to PLOS CB.docx

# Peer review: Decision Letter 1

**DOI:** 10.1371/journal.pcbi.1014389.r003

**Authors:** Lun Hu, Denise Kühnert

1 Jun 2026

Dear Dr. Utkarsh,

We are pleased to inform you that your manuscript 'A new method for augmenting short time series, with application to pain events in sickle cell disease' has been provisionally accepted for publication in PLOS Computational Biology.

Before your manuscript can be formally accepted you will need to complete some formatting changes, which you will receive in a follow up email. A member of our team will be in touch with a set of requests.

Please note that your manuscript will not be scheduled for publication until you have made the required changes, so a swift response is appreciated.

IMPORTANT: The editorial review process is now complete. PLOS will only permit corrections to spelling, formatting or significant scientific errors from this point onwards. Requests for major changes, or any which affect the scientific understanding of your work, will cause delays to the publication date of your manuscript.

Should you, your institution's press office or the journal office choose to press release your paper, you will automatically be opted out of early publication. We ask that you notify us now if you or your institution is planning to press release the article. All press must be co-ordinated with PLOS.

Thank you again for supporting Open Access publishing; we are looking forward to publishing your work in PLOS Computational Biology.

Best regards,

Lun Hu

Academic Editor

PLOS Computational Biology

Denise Kühnert

Section Editor

PLOS Computational Biology

***********************************************************

All reviewers were satisfied with the changes made in this revised version of the manuscript.

Reviewer's Responses to Questions

**Comments to the Authors:**

**Please note here if the review is uploaded as an attachment.**

Reviewer #1: The authors changes and responses to my comments are very good. The addition of S4 is really excellent.

Reviewer #2: The manuscript has been well revised and is recommended for acceptance.

Reviewer #3: Authors have revised the manuscript according to the suggestions of the reviewer.

The present version may be accepted.

**********

**Have the authors made all data and (if applicable) computational code underlying the findings in their manuscript fully available?**

The PLOS Data policy requires authors to make all data and code underlying the findings described in their manuscript fully available without restriction, with rare exception (please refer to the Data Availability Statement in the manuscript PDF file). The data and code should be provided as part of the manuscript or its supporting information, or deposited to a public repository. For example, in addition to summary statistics, the data points behind means, medians and variance measures should be available. If there are restrictions on publicly sharing data or code —e.g. participant privacy or use of data from a third party—those must be specified.

Reviewer #1: Yes

Reviewer #2: None

Reviewer #3: None

**********

PLOS authors have the option to publish the peer review history of their article (what does this mean?). If published, this will include your full peer review and any attached files.

If you choose “no”, your identity will remain anonymous but your review may still be made public.

**Do you want your identity to be public for this peer review?**   For information about this choice, including consent withdrawal, please see our Privacy Policy.

Reviewer #1: No

Reviewer #2: No

Reviewer #3: No

# Editor report: Acceptance letter

**DOI:** 10.1371/journal.pcbi.1014389.r004

**Authors:** Lun Hu, Denise Kühnert

PCOMPBIOL-D-26-00088R1

A new method for augmenting short time series, with application to pain events in sickle cell disease

Dear Dr Utkarsh,

I am pleased to inform you that your manuscript has been formally accepted for publication in PLOS Computational Biology. Your manuscript is now with our production department and you will be notified of the publication date in due course.

The corresponding author will soon be receiving a typeset proof for review, to ensure errors have not been introduced during production. Please review the PDF proof of your manuscript carefully, as this is the last chance to correct any errors. Please note that major changes, or those which affect the scientific understanding of the work, will likely cause delays to the publication date of your manuscript.

Soon after your final files are uploaded, unless you have opted out, the early version of your manuscript will be published online. The date of the early version will be your article's publication date. The final article will be published to the same URL, and all versions of the paper will be accessible to readers.

For Research, Software, and Methods articles, you will receive an invoice from PLOS for your publication fee after your manuscript has reached the completed accept phase. If you receive an email requesting payment before acceptance or for any other service, this may be a phishing scheme. Learn how to identify phishing emails and protect your accounts at https://explore.plos.org/phishing.

Thank you again for supporting PLOS Computational Biology and open-access publishing. We are looking forward to publishing your work!

With kind regards,

Anita Estes

PLOS Computational Biology | Carlyle House, Carlyle Road, Cambridge CB4 3DN | United Kingdom ploscompbiol@plos.org | Phone +44 (0) 1223-442824 | ploscompbiol.org | @PLOSCompBiol
